# The persistence of very low correlations between NIH research funding and disease burdens

**DOI:** 10.1016/j.puhip.2024.100580

**Published:** 2024-12-21

**Authors:** Ashley J.R. Carter, Milena Gevorkian

**Affiliations:** California State University Long Beach, Long Beach, CA, USA

**Keywords:** Research funding, Disease burden, Disability adjusted life years, Years of life lost

## Abstract

**Objectives:**

The degree to which the allocation of disease-specific research funding by the NIH is proportional to disease burden is an important question. This study examined the historical relationship between NIH funding allocation and disease burden for a variety of medical conditions.

**Study design:**

Coefficients of relatedness for the linear relationships between funding and disease burden for 27 medical conditions over a period exceeding twenty years were calculated.

**Methods:**

Publicly available data from 2009 to 2019, and previously published data from 1994 to 2004, was obtained to compare disease-specific research funding from the NIH to burden of disease values (mortality, prevalence, incidence, DALYs, and YLLs) for 27 diseases.

**Results:**

We identified very weak and declining correlations (e.g., R^2^ < 0.03) between funding and the five measures of burden for the 27 diseases. The weak relationships persist even when HIV/AIDS is omitted (e.g., R^2^ < 0.1). A recent decline in the overall strengths of the funding burden relationships is attributable to novel investment in Alzheimer's disease research.

**Conclusions:**

The weak correlations reveal long-standing inefficiencies in the NIH disease funding allocation process. The recent increased and focused funding for Alzheimer's disease may not be justified by an objective analysis which considers disease burdens. Increased efficiency of medical research may be realized by improving the poor match between disease burden and funding allocation.

## Graphical abstract

**Figure:**
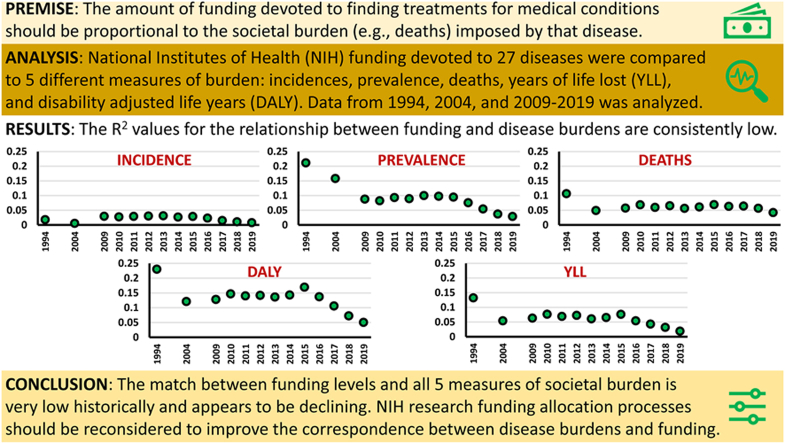

## 1 Introduction

The National Institutes of Health (NIH) operate with a mandate to collect crucial knowledge regarding the natural and behavioral aspects of living organisms and in turn apply such discoveries to enhance all aspects of health [1]. Ranking first among public agencies in biomedical research funding, the NIH has a multibillion-dollar budget ranging from approximately $12 billion in 1996 to over $45 billion in 2022 [2]. In the 1990s, concerns regarding funding allocations as they relate to disease burden were raised, causing Congress to order a reassessment of the allocation process itself. This resulted in a recommendation that measures should be taken to reallocate funds according to disease-specific factors, including the burden imposed on society from each disease [3,4].

Although such a reallocation process took place, the degree to which this addressed the issue is unclear. A subsequent study [5] identified discrepancies in the allocation process by examining 29 health conditions as they related to multiple measures of the burden of disease. These measures included: incidence of the disease, prevalence of the disease, mortality rates, years of life lost (YLLs), and Disability-adjusted life years (DALYs). Each measure had either no correlation with funding or a very weak one. DALYs were identified as the measure showing the most consistent relationship with funding, but this was weak and a strong correlation was not proven nor established [5].

In 2006, the National Institutes of Health Reform Act reiterated the importance of battling hardships related to health by recommending prioritization of funding allocations based on disease specific burdens to society [4,6]. To our knowledge, the most recent studies of the relationship between overall NIH funding and multiple measures of the burden of disease are over 10 years old [4,7] although there has been a recent analysis which focused exclusively on DALYs [8].

It remains unclear whether the current NIH funding allocation process accurately reflects a full set of measures of the burden of disease. It is also unclear whether the match between societal burden and funding allocation has been improving or worsening over the past two decades. We therefore examined five disease burden metrics and levels of funding by the NIH for 27 medical conditions for the years 2009 through 2019 and compared them to previously reported values for 1994 and 2004 to investigate these questions. Our initial expectations are that there will be significant relationships between the health care burdens and funding allocations as suggested by the 2006 National Institutes of Health Reform Act, with the strongest relationships predicted for mortalities and YLLs.

## 2 Methods

We calculated the United States’ burden of disease and obtained National Institutes of Health levels of funding from 2009 to 2019 for 27 disease categories. Comparable values from two prior studies were obtained from the literature: one comparing funding data from 1996 and disease burden from 1994 [5] and one comparing funding data from 2006 to disease burden from 2004 [4]. Our methods followed those used in the earlier studies.

### 2.1 Sources of data

United States disease burden data for the years 2009 through 2019 was collected from the Institute for Health Metrics and Evaluation (IHME), Global Burden of Disease (GBD) Database [9]. The IHME, more specifically the Global Health Data Exchange, provides a vast array of global health data that is up to date and relevant to crucial studies concerning the burden of disease [9]. Disease-specific burden data for 1994 and 2004 were taken from two previously published papers [4,5]. We used data for 27 diseases based on availability in the current data source and direct comparability with those reported in two previously published papers [4,5].

We analyzed five measures of disease-specific burden: incidence, prevalence, mortality, years of life lost (YLLs), and disability adjusted life years (DALYs). YLLs for each disease were calculated by multiplying the standardized life expectancy at the specific age the death took place by the estimated number of deaths [10]. DALYs for each disease were calculated by summing the YLL value and the number of years lived with a disability discounted by an established value [11].

The amounts of disease specific funding by the NIH were retrieved from the Research, Condition, and Disease Categories (RCDC) Funding Summary and include data from 2008 to 2019 [12]. Funding data for 1996 and 2006 were taken from two previously published papers [4,5]. Exact matches between the narrow categories for a specific disease or condition in both data sets were used, no data for broader categories was used (e.g., no comparisons of funding for a broad category applied to subset of diseases within that category)

### 2.2 Calculations

Disease burden data for prevalence, incidence, mortality, DALYs, and YLLs were initially obtained or calculated as raw values across 27 disease types from 2009 to 2019. These raw values were converted to percentages of the overall burden by dividing each disease burden value by the sum of the set of burden values. Subsequent calculations were performed using these percentage values.

Raw and percentage funding NIH values for the 27 disease types were calculated using the same procedure.

To estimate the strength of the relationship between the variables, we calculated the correlation coefficients and coefficients of determination, r and R^2^, for the five disease burdens and their respective funding levels across all 27 diseases for the years 1994/6, 2004/6, and 2009–2019.

A sensitivity analysis was conducted to identify any diseases that had a disproportionate effect on R^2^ values to identify outliers within the data that substantially increase or decrease the R^2^ values via their inclusion or exclusion. Each of the conditions was individually removed from the data sets and the R^2^ values calculated from a new set of percentages with the condition omitted. For the vast majority of conditions, the effects of inclusion or exclusion on the R^2^ values were minimal (e.g., values changed by less than 0.01). Inclusion or exclusion of HIV/AIDS had a dramatic effect across all burdens due to its very low burden and very high funding levels, reducing the R^2^ dramatically in all cases. Inclusion or exclusion of dental issues showed the next largest effect, but this was relatively minimal. We therefore excluded HIV/AIDS from our primary reported analyses, but retained dental issues. Analyses which include HIV/AIDS or exclude both HIV/AIDS and dental issues are available in the Supplementary Materials.

Unless otherwise stated, results described are for the 26 remaining disease types.

## 3 Results

**Fig. 1:**
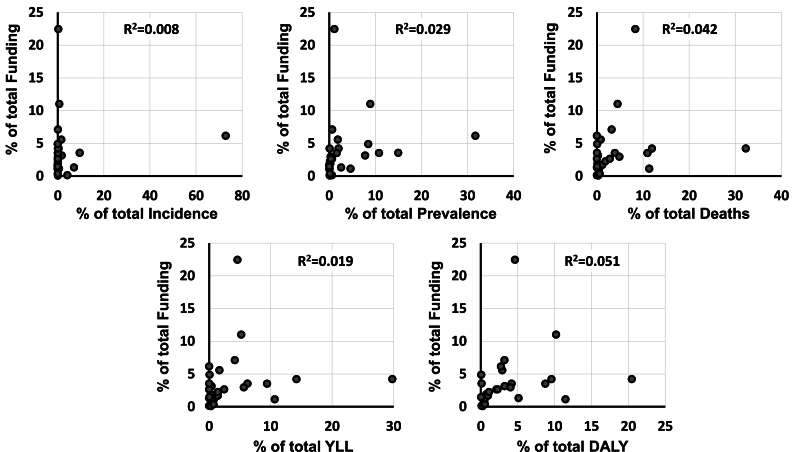
Relationships Between Funding and Disease Burden in 2019. Plots show data for five measures of disease burden (incidence, prevalence, deaths, YLLs, and DALYs) and the NIH funding designated for 26 medical conditions for the year 2019. The percentages of the overall burden and funding are plotted for each condition.

**Fig. 2:**
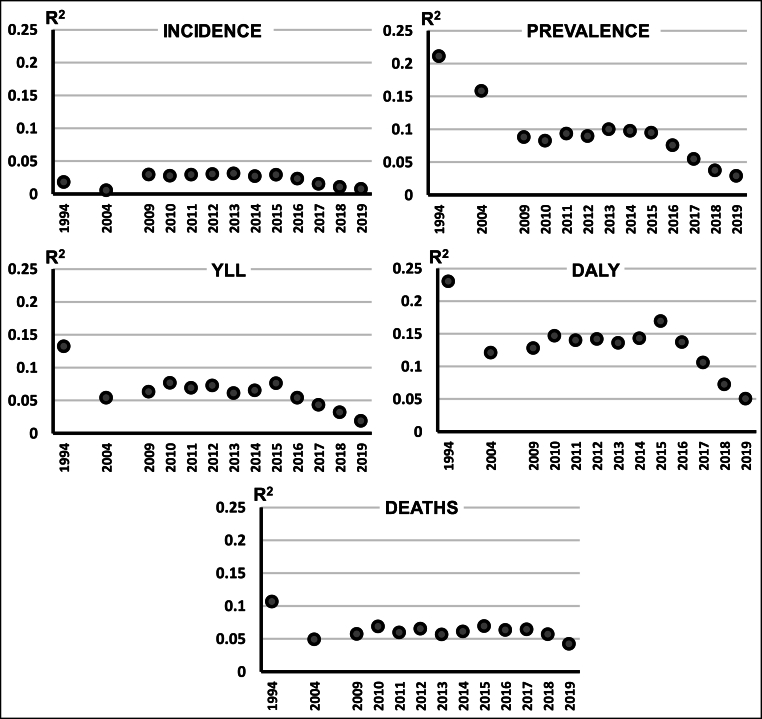
Relationships between funding and disease burden over time. Plot of R^2^ values based on 26 diseases for the relationship between measures of five measures of disease burden (incidence, prevalence, deaths, YLL, and DALY) and NIH funding for the years 1994, 2004, and 2009–2019.

The coefficient of determination values indicated weak correlations between the 26 disease-specific NIH funding levels and all five categories of disease-specific burden. For example, using the data from 2019, the R^2^ values ranged from a low of 0.008 to a high of 0.051 (Fig. 1). Generally low values were seen across the entire range of years examined (Fig. 2).

When examining the different burdens across all years, the R^2^ for DALYs and funding was the highest, albeit with an average value of approximately 0.15 which indicates a weak correlation between these factors. The next largest set of R^2^ values were seen for prevalence vs funding, with values averaging approximately 0.1. The R^2^ values for incidence, YLL, and total deaths vs funding were below 0.1 for almost all years examined.

If HIV/AIDS is included in the analysis, the R^2^ values for all years and burdens are much lower, with the R^2^ values for every year and condition dropping to less than 0.02 except for YLL in 1994/6 which is 0.11 (see Supplementary Materials). If dental issues are removed the R^2^ values change slightly (increasing for DALYs, Deaths, and YLL, but decreasing for incidence and prevalence) with no values changing by more than 0.03 except for prevalence in 2004 (see Supplementary Materials).

## 4 Discussion

Our analysis, excluding HIV, reveals persistently weak observed correlations between the disease burden categories and disease-specific NIH funding values lasting for over 20 years. Of the five disease burden categories examined, DALYs demonstrated the highest correlation with funding levels. Although these values were the highest over the years, they remained at a low magnitude and are indicative of a long-term weak relationship.

The overall statistical significance of the observed correlations between disease burdens and funding was extremely low. For the DALYs, in only 9 of the 13 years examined were the correlation coefficients statistically significant and the R^2^ values ranged from a high of 0.230 to a low of 0.050 with a mean value of only 0.132. For the other four disease burdens and 13 years examined, representing 62 comparisons, only one correlation coefficient was significant (prevalence in 1994) and the R^2^ values were mostly (48 of 52) below 0.1 in magnitude with mean values of 0.093, and 0.063, 0.063, and 0.022 for prevalence, YLL, Deaths, and incidence respectively.

The highest magnitude correlations appeared in earlier years with a subsequent pattern of stasis or general decline in the consistency of the observed relationships between disease burden and NIH research funding (Fig. 2). To the extent that there is convincing evidence of any relationship between disease burden and research funding, this relationship seems to be staying the same or getting weaker over time.

The sensitivity analysis caused us to remove HIV from our disease burden list in our analyses. The inclusion of HIV in our analyses would result in no significant correlation coefficients (t-tests of all 65 values give a minimum of p value of 0.2 with a mean of 0.807) and R^2^ values ranging from a high of 0.065 to a low of 1.86 x 10^−5^ with a mean value of only 0.0038. Our aforementioned results excluding HIV are therefore somewhat conservative and overestimate the strength of the relationship between overall disease burdens and research funding.

In totality, our results suggest that during the period from 1994 to 2019 the allocation of NIH research funding to different diseases was not related to the levels of disease incidence, prevalence, mortality, or YLL with weak evidence it they may have been slightly related to DALYs.

Given that most disease burden factors were barely related to funding, with none of them showing a strong relationship, it seems that policy makers may be focusing on only one factor of disease burden, DALYs, rather than considering all factors [4,5]. It therefore seems that the goal of the National Institutes of Health Reform Act has not been realized since the relationships between funding and disease burden are mostly nonexistent.

There are a few potential limitations or weaknesses of the data and methods which we've used.

One limitation is that the data is confined strictly to the National Institutes of Health spending in United States, limiting broad claims about the relationships between funding and research across the myriad agencies that conduct health care research. While the NIH in the US is the largest single agency making allocation decisions, relative burdens in US are likely to differ greatly from those in other nations and the global average. This problem is particularly acute in the developing world [13], but it can also be seen in European countries [14]. While this concern has been described, attention to this problem is limited and worthy of more consideration [15,16].

Another possible weakness of this approach is the built-in lags between research being conducted and health care innovations having measurable effects. Lags between research and the health care improvements coming later are inevitable and essentially impossible to predict. Lags between changes in disease burden and research allocation are unavoidable, but much more amenable to minimization if disease burden is regularly monitored and used to update funding allocations. For example, the major progress made in treating breast cancer without subsequent reallocation of those research resources to other diseases may explain the similar pattern of over-funding on research for this disease in both the US and the UK [17].

Nevertheless, our evidence does strongly suggest that the relative disease burdens for these 26 (or 27) diseases, as measured by the five metrics examined, do not seem influential in determining the NIH allocations to research funding, this raises the following question: what other factors determine NIH research allocations to diseases?

Our analysis shows that HIV receives the highest funding, relative to burden, of all diseases. Whether this is tied to ancillary benefits such research may present to the NIH itself is an open question. In support of this idea, HIV research has been tied to scientific breakthroughs for other serious illnesses. HIV treatments such as the use of protease inhibitors and nucleoside polymerase inhibitors, have paved the path for similar treatments for hepatitis C [18]. Discoveries concerning the immune system have been made via HIV research, thus allowing for development of new therapeutic alternatives, decreases in drug costs, and the production of generic medicines [18]. In addition, HIV research has allowed for the further development of treatments and cures for cardiovascular diseases and even cancers [18]. The high rate of funding for HIV/AIDs may therefore be associated with its key role in immunology studies and perceived benefits for other disease categories.

The extremely high economic costs HIV presents to the healthcare system may serve as an additional reason why the NIH allocates such a high degree of funding for this disease. Individuals living with HIV/AIDS may be considered disabled when meeting specific criteria provided by the Social Security Administration, granting them the right to receive Social Security Disability Insurance and Supplemental Security Income [19]. This additional government cost associated with eligibility for government funded support may be related to motivation for high NIH funding, whereas other diseases, despite their high burden, may not impose similar amounts of economic costs via long-term disability benefits.

There is some additional evidence that economic factors, rather than purely health related ones, drive research funding. A 2013 study found that funding values were strongly related to specific disease categories including deaths and hospital admissions, but failed to display a significant relationship with other crucial categories such as DALYs and YLLs [20]. A 2012 analysis of NIH research funding for types of cancer appeared more associated with Medicare expenditures, national health care costs, and productivity losses than with mortalities, DALYs, or YLL [21].

We observed that the highest R^2^ values tended to be found in the earliest years in our data, suggesting that original allocations may have initially been made based upon the criteria examined in our study, but a lack of allocation updating reduced the correlations as disease burdens changed. Indeed, a prior study [8] found that NIH funding allocations for a majority of disease categories in 2019 were similar to those granted 10 years prior, completely disregarding changes in disease burden. It is quite possible that initial allocation processes may have more appropriately matched disease burden with NIH funding, but the updating of allocations to disease burden categories was neglected and the match therefore declined over time as advancements are made.

Declines in the R^2^ values may also be caused by targeted funding decisions unrelated to relative burden and possibly even politically motivated. This seems to account for the apparent decline beginning in 2016. We analyzed the data for the cause of this decline and determined that while the magnitudes of the relative disease burdens for the category “Alzheimer's Disease and Other Dementias” were unchanged, the relative funding increased considerably. This was a deliberate political act; in 2016 the United States Government increased the amount of spending allocated towards Alzheimer's research with a targeted increase of approximately 5 % of the overall NIH budget [22]. Being designed by politicians, individuals who use metrics other than public health to make funding decisions, this targeted funding was unrelated to the relative disease burden of this single category. In fact, due to the sudden nature of this increase, the NIH faced issues concerning overburdened NIH staff and some researchers adding reference to Alzheimer's disease to their barely related grants to take advantage of the increased funding [22]. This decision-making in the absence of proper consideration appears to have exacerbated the pre-existing inefficient allocation of limited funding resources.

We also can't overlook the possibility that social and cultural factors within the research community may be at play, especially with regard to maintaining relative allocation levels. It's possible that personal opinions and preferences among influential NIH staff members could play a role in the allocation processes, favoring persistent idiosyncratic or irrelevant funding strategies. Additionally, at the researcher level, successful laboratories which receive more funding may continue to receive funding over the entire career of the PI whereas newer laboratories studying more novel or newly increasing burdens struggle to reach this level. Peer reviews from external parties tend to fixate on scientific merit in addition to the burden of disease and papers with well-established authors are more likely to be accepted by journals than those of identical quality by less-established authors [23]. These two processes favor established research programs which continue to examine the same system or disease regardless of changes to societal burden. Such factors likely contribute to the growing mismatch between societal burden and NIH funding.

The continued study and research of specific diseases, despite the development of effective treatment methods, can therefore account for the development of over/under funding of disease categories. Basic and clinical research is crucial in discovering new and innovative means in which diseases can be stabilized or treated, but our results suggest that a culture of research program inertia may act against this.

We therefore suggest that research funding allocation processes be reconsidered to provide a better correspondence between the disease burden and funding each condition receives. Given the limitations to total funding, more efficient funding allocation processes may provide the best mechanism to reduce the societal burdens arising from disease.

## Ethical approval

Not applicable.

## Competing interests

None.

## Financial disclosure

no funding received for project.

## Conflict of interest

none.
