# Supplementary material for: The persistence of very low correlations between NIH research funding and disease burdens
Source: Public Health Pract (Oxf). 2024 Dec 21;9:100580. doi: 10.1016/j.puhip.2024.100580 (PMC11754078; doi:10.1016/j.puhip.2024.100580)
Supplement: Multimedia component 1 [file mmc1.pdf]

Supplementary Appendix

Cover page	1
Figures	2
Data tables	7
References	59

Ashley J.R. Carter, Ph.D[1]
Milena Gevorkian, BS[1]

1. California State University Long Beach, Long Beach, CA

Figures

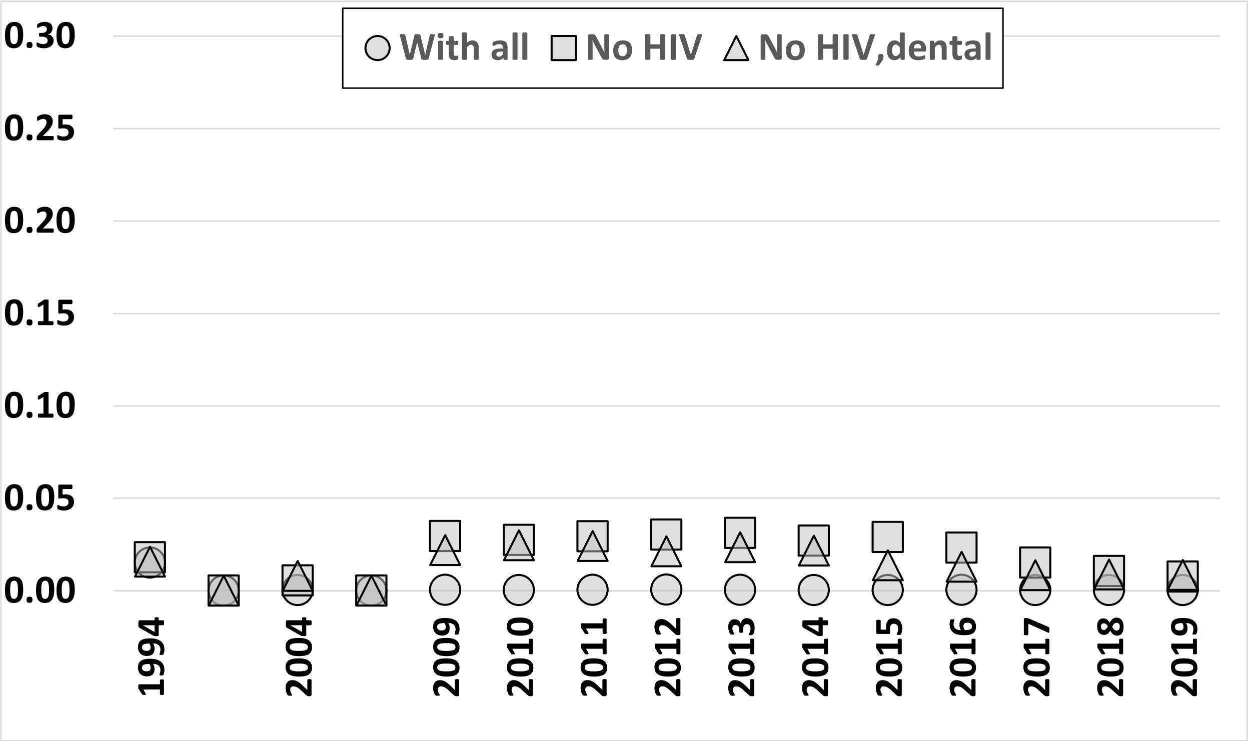

Complete data for incidence data. Incidence data analogous to figure 2 from paper showing R^2 values over time for all 27 diseases ('With all"), 26 diseases (excluding HIV/AIDS as in paper, "No HIV"), and 25 diseases (excluding HIV/AIDS and dental issues).

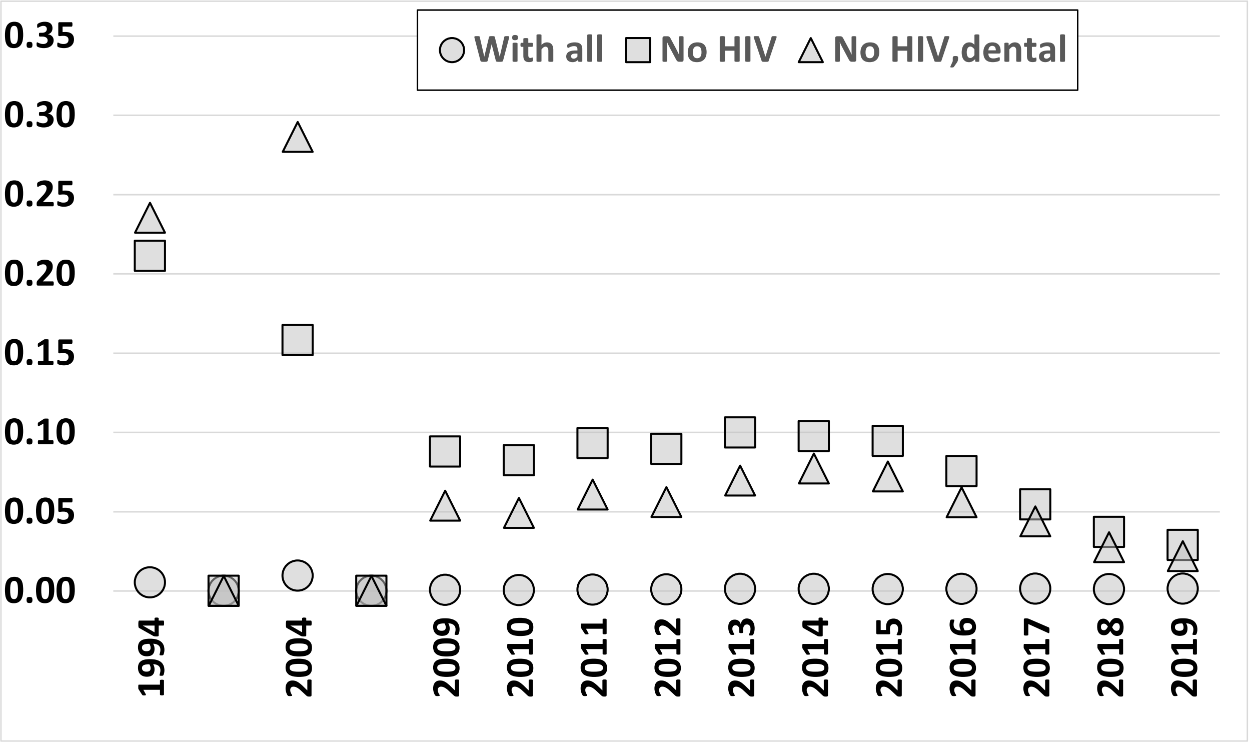

Complete data for prevalence data. Incidence data analogous to figure 2 from paper showing R^2 values over time for all 27 diseases ('With all"), 26 diseases (excluding HIV/AIDS as in paper, "No HIV"), and 25 diseases (excluding HIV/AIDS and dental issues).

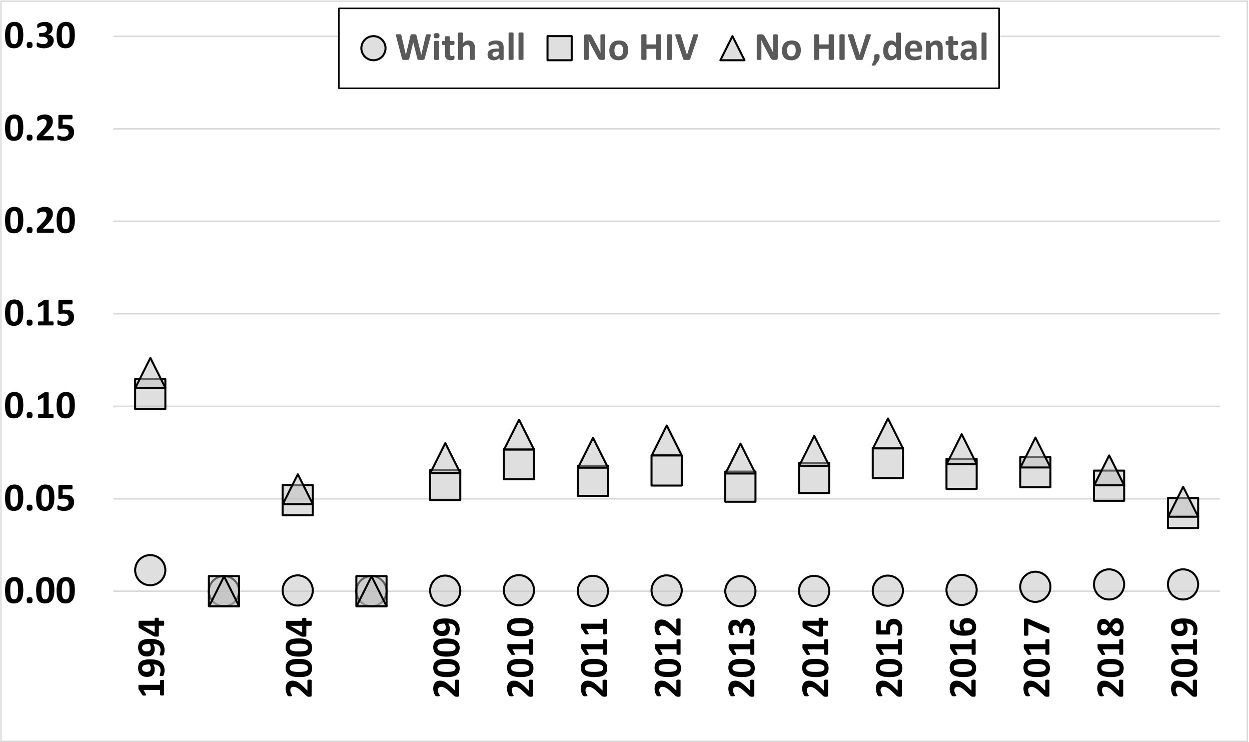

Complete data for deaths data. Deaths data analogous to figure 2 from paper showing R^2 values over time for all 27 diseases ('With all"), 26 diseases (excluding HIV/AIDS as in paper, "No HIV"), and 25 diseases (excluding HIV/AIDS and dental issues).

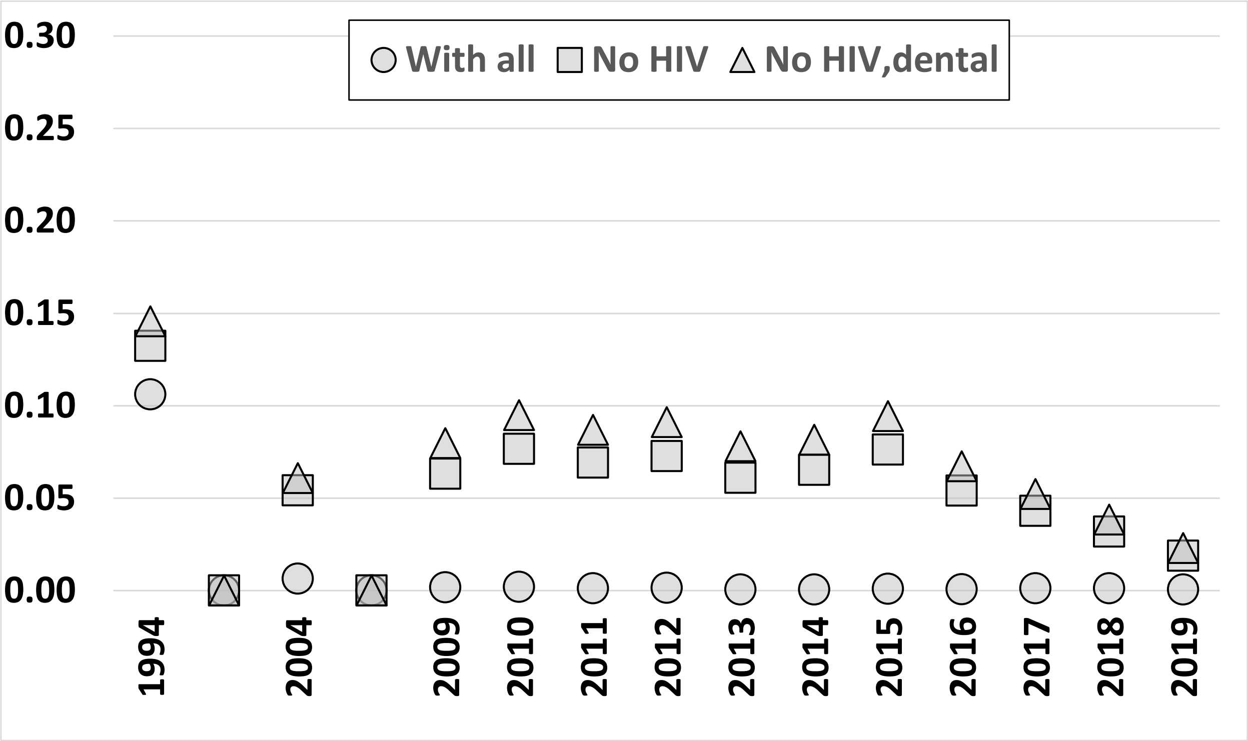

Complete data for YLL data. YLL data analogous to figure 2 from paper showing R^2 values over time for all 27 diseases ('With all"), 26 diseases (excluding HIV/AIDS as in paper, "No HIV"), and 25 diseases (excluding HIV/AIDS and dental issues).

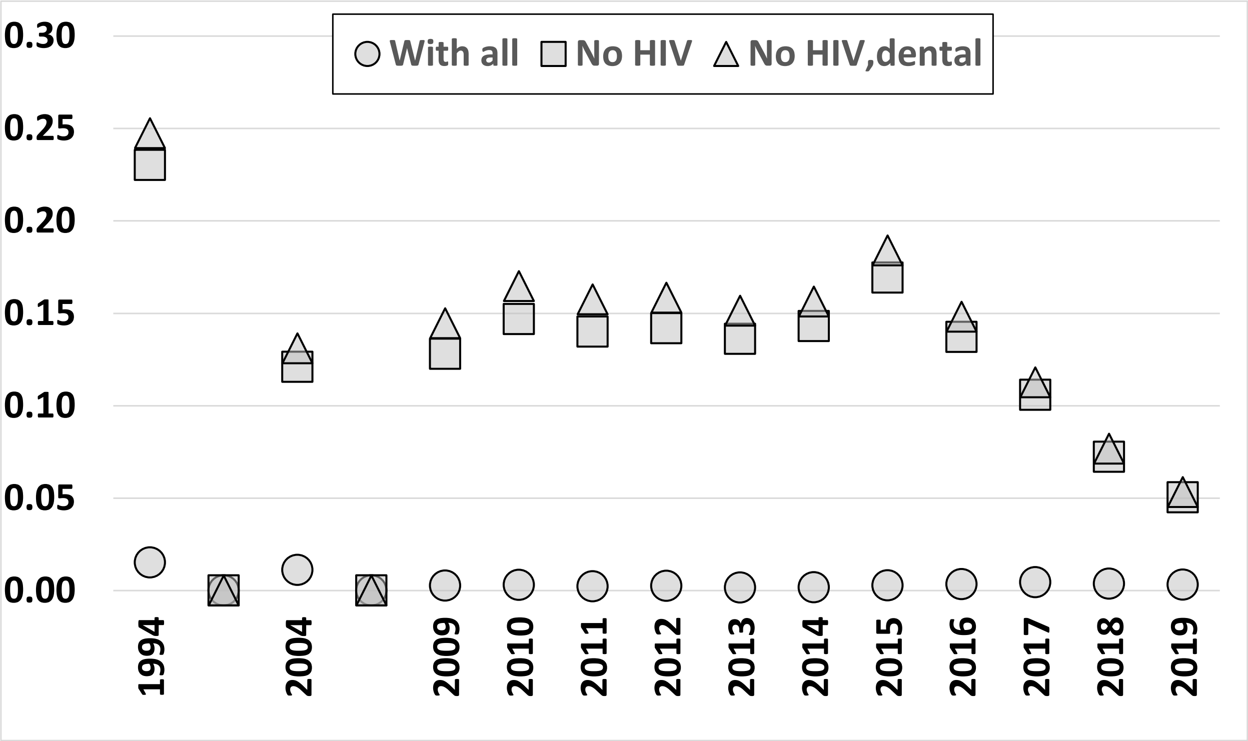

Complete data for DALY data. DALY data analogous to figure 2 from paper showing R^2 values over time for all 27 diseases ('With all"), 26 diseases (excluding HIV/AIDS as in paper, "No HIV"), and 25 diseases (excluding HIV/AIDS and dental issues).

Data Tables

CAUSES-2009	DALYs	Incidence	Deaths	Prevalence	YLL	Funding (M)
Alcohol use disorders	1160572	3422251	11476	7512882	425807	$441
Alzheimer's disease and other dementias	1676594	531308	118965	3963342	1111910	$457
Asthma	1145573	3512349	3813	26663320	114714	$284
Breast cancer	1298811	230439	48865	2477636	1126304	$722
Cervical cancer	200577	17100	6815	101729	192383	$84
Chronic obstructive pulmonary disease	4158767	1111562	155906	16895294	2455087	$96
Cirrhosis and other chronic liver diseases	1570605	87021	54496	38746921	1557751	$274
Colon and rectum cancer	1478938	186706	69871	1117006	1380674	$281
Diabetes mellitus	3612087	1353142	71235	29160902	1411156	$1,030
HIV/AIDS	547636	45659	10551	1332568	437558	$3,019
Idiopathic epilepsy	354937	125918	1888	1212798	74766	$128
Ischemic heart disease	8047039	960832	495676	7800444	7799315	$426
Major depressive disorder	2315044	16894856	0	11545195	0	
Multiple sclerosis	188711	9808	3414	364677	98323	$137
Oral disorders	830242	160148921	0	113133149	0	$490
Otitis media	47903	9082008	23	2461106	764	$15
Ovarian cancer	400067	25315	17745	100183	386855	$102
Parkinson's disease	374953	63840	24760	478453	310142	$162
Peptic ulcer disease	95148	177215	4486	416214	76178	$17
Pneumoconiosis	19712	9614	991	35572	14514	$108
Prostate cancer	719708	234836	37724	2077018	555229	$310
Schizophrenia	988950	73699	0	1580785	0	$265
Sexually transmitted infections excluding HIV	44976	21201417	157	61290188	3766	$250
Stroke	3277070	412535	159400	6082476	2350822	$329
Tracheal, bronchus, and lung cancer	3821663	227025	182650	397041	3765908	$178
Tuberculosis	24178	10548	891	35964828	20188	$189
Uterine cancer	181926	58054	7511	455609	152054	$25

Raw data values for 2009 (all disorders).

CAUSES-2009	DALYs	Incidence	Deaths	Prevalence	YLL	Funding
Alcohol use disorders	3.008	1.554	0.771	2.012	1.649	4.491
Alzheimer's disease and other dementias	4.345	0.241	7.988	1.062	4.306	4.654
Asthma	2.969	1.595	0.256	7.141	0.444	2.892
Breast cancer	3.366	0.105	3.281	0.664	4.362	7.353
Cervical cancer	0.520	0.008	0.458	0.027	0.745	0.855
Chronic obstructive pulmonary disease	10.779	0.505	10.468	4.525	9.508	0.978
Cirrhosis and other chronic liver diseases	4.071	0.040	3.659	10.378	6.033	2.791
Colon and rectum cancer	3.833	0.085	4.692	0.299	5.347	2.862
Diabetes mellitus	9.362	0.614	4.783	7.810	5.465	10.490
HIV/AIDS	1.419	0.021	0.708	0.357	1.695	30.747
Idiopathic epilepsy	0.920	0.057	0.127	0.325	0.290	1.304
Ischemic heart disease	20.857	0.436	33.282	2.089	30.204	4.339
Major depressive disorder	6.000	7.672	0.000	3.092	0.000	0.000
Multiple sclerosis	0.489	0.004	0.229	0.098	0.381	1.395
Oral disorders	2.152	72.724	0.000	30.301	0.000	4.990
Otitis media	0.124	4.124	0.002	0.659	0.003	0.153
Ovarian cancer	1.037	0.011	1.191	0.027	1.498	1.039
Parkinson's disease	0.972	0.029	1.662	0.128	1.201	1.650
Peptic ulcer disease	0.247	0.080	0.301	0.111	0.295	0.173
Pneumoconiosis	0.051	0.004	0.067	0.010	0.056	1.100
Prostate cancer	1.865	0.107	2.533	0.556	2.150	3.157
Schizophrenia	2.563	0.033	0.000	0.423	0.000	2.699
Sexually transmitted infections excluding HIV	0.117	9.628	0.011	16.416	0.015	2.546
Stroke	8.494	0.187	10.703	1.629	9.104	3.351
Tracheal, bronchus, and lung cancer	9.905	0.103	12.264	0.106	14.584	1.813
Tuberculosis	0.063	0.005	0.060	9.633	0.078	1.925
Uterine cancer	0.472	0.026	0.504	0.122	0.589	0.255

Percentage data values for 2009 (all disorders).

CAUSES-2009	DALYs	Incidence	Deaths	Prevalence	YLL	Funding
Alcohol use disorders	3.051	1.554	0.776	2.019	1.677	6.485
Alzheimer's disease and other dementias	4.408	0.241	8.045	1.065	4.380	6.721
Asthma	3.012	1.595	0.258	7.167	0.452	4.176
Breast cancer	3.415	0.105	3.304	0.666	4.437	10.618
Cervical cancer	0.527	0.008	0.461	0.027	0.758	1.235
Chronic obstructive pulmonary disease	10.934	0.505	10.543	4.541	9.672	1.412
Cirrhosis and other chronic liver diseases	4.129	0.040	3.685	10.415	6.137	4.029
Colon and rectum cancer	3.888	0.085	4.725	0.300	5.439	4.132
Diabetes mellitus	9.497	0.615	4.817	7.838	5.559	15.147
Idiopathic epilepsy	0.933	0.057	0.128	0.326	0.295	1.882
Ischemic heart disease	21.157	0.436	33.520	2.097	30.725	6.265
Major depressive disorder	6.087	7.674	0.000	3.103	0.000	0.000
Multiple sclerosis	0.496	0.004	0.231	0.098	0.387	2.015
Oral disorders	2.183	72.739	0.000	30.409	0.000	7.206
Otitis media	0.126	4.125	0.002	0.662	0.003	0.221
Ovarian cancer	1.052	0.011	1.200	0.027	1.524	1.500
Parkinson's disease	0.986	0.029	1.674	0.129	1.222	2.382
Peptic ulcer disease	0.250	0.080	0.303	0.112	0.300	0.250
Pneumoconiosis	0.052	0.004	0.067	0.010	0.057	1.588
Prostate cancer	1.892	0.107	2.551	0.558	2.187	4.559
Schizophrenia	2.600	0.033	0.000	0.425	0.000	3.897
Sexually transmitted infections excluding HIV	0.118	9.630	0.011	16.474	0.015	3.676
Stroke	8.616	0.187	10.779	1.635	9.261	4.838
Tracheal, bronchus, and lung cancer	10.048	0.103	12.352	0.107	14.835	2.618
Tuberculosis	0.064	0.005	0.060	9.667	0.080	2.779
Uterine cancer	0.478	0.026	0.508	0.122	0.599	0.368

Percentage data values for 2009 (HIV/AIDS removed).

CAUSES-2009	DALYs	Incidence	Deaths	Prevalence	YLL	Funding
Alcohol use disorders	3.119	5.702	0.776	2.902	1.677	6.989
Alzheimer's disease and other dementias	4.506	0.885	8.045	1.531	4.380	7.242
Asthma	3.079	5.852	0.258	10.299	0.452	4.501
Breast cancer	3.491	0.384	3.304	0.957	4.437	11.442
Cervical cancer	0.539	0.028	0.461	0.039	0.758	1.331
Chronic obstructive pulmonary disease	11.178	1.852	10.543	6.526	9.672	1.521
Cirrhosis and other chronic liver diseases	4.222	0.145	3.685	14.966	6.137	4.342
Colon and rectum cancer	3.975	0.311	4.725	0.431	5.439	4.453
Diabetes mellitus	9.709	2.255	4.817	11.263	5.559	16.323
Idiopathic epilepsy	0.954	0.210	0.128	0.468	0.295	2.029
Ischemic heart disease	21.629	1.601	33.520	3.013	30.725	6.751
Major depressive disorder	6.222	28.149	0.000	4.459	0.000	0.000
Multiple sclerosis	0.507	0.016	0.231	0.141	0.387	2.171
Otitis media	0.129	15.132	0.002	0.951	0.003	0.238
Ovarian cancer	1.075	0.042	1.200	0.039	1.524	1.616
Parkinson's disease	1.008	0.106	1.674	0.185	1.222	2.567
Peptic ulcer disease	0.256	0.295	0.303	0.161	0.300	0.269
Pneumoconiosis	0.053	0.016	0.067	0.014	0.057	1.712
Prostate cancer	1.934	0.391	2.551	0.802	2.187	4.913
Schizophrenia	2.658	0.123	0.000	0.611	0.000	4.200
Sexually transmitted infections excluding HIV	0.121	35.324	0.011	23.673	0.015	3.962
Stroke	8.808	0.687	10.779	2.349	9.261	5.214
Tracheal, bronchus, and lung cancer	10.272	0.378	12.352	0.153	14.835	2.821
Tuberculosis	0.065	0.018	0.060	13.891	0.080	2.995
Uterine cancer	0.489	0.097	0.508	0.176	0.599	0.396

Percentage data values for 2009 (HIV/AIDS and Oral disorders removed).

CAUSES-2010	DALYs	Incidence	Deaths	Prevalence	YLL	Funding
Alcohol use disorders	1178234	3459928	11831	7586423	436859	454
Alzheimer's disease and other dementias	1710584	542968	121781	4050427	1134042	450
Asthma	1190790	3664871	3730	27947243	111618	244
Breast cancer	1288770	229746	48781	2507175	1115151	763
Cervical cancer	201420	17101	6893	101487	193236	93
Chronic obstructive pulmonary disease	4221325	1136382	158405	17247313	2484572	118
Cirrhosis and other chronic liver diseases	1588413	87657	55472	39600811	1575508	284
Colon and rectum cancer	1487679	188816	70297	1138403	1387901	291
Diabetes mellitus	3682172	1406555	69915	30473675	1380695	1046
HIV/AIDS	488804	48175	9143	1362866	374158	3085
Idiopathic epilepsy	358205	126571	1923	1227943	75310	134
Ischemic heart disease	7925754	946542	490097	7825514	7673145	457
Major depressive disorder	2335808	17051167	0	11654463	0	
Multiple sclerosis	190642	9885	3463	370314	98902	133
Oral disorders	832078	161334861	0	113922888	0	497
Otitis media	48092	9009449	23	2469189	764	19
Ovarian cancer	389985	24576	17343	97233	377151	122
Parkinson's disease	382119	66751	25309	489090	315902	154
Peptic ulcer disease	94202	181498	4421	426586	74765	30
Pneumoconiosis	19283	9975	967	36027	14021	93
Prostate cancer	728536	240795	38084	2132089	560504	331
Schizophrenia	995781	74104	0	1592992	0	276
Sexually transmitted infections excluding HIV	44798	21219979	155	60946947	3690	250
Stroke	3274708	417878	158981	6238298	2327216	337
Tracheal, bronchus, and lung cancer	3776579	225567	181440	398554	3721057	201
Tuberculosis	23453	10137	868	36319552	19507	189
Uterine cancer	187805	60371	7741	475035	156747	26

Raw data values for 2010 (all disorders).

CAUSES-2010	DALYs	Incidence	Deaths	Prevalence	YLL	Funding
Alcohol use disorders	3.049	1.560	0.796	2.004	1.706	4.505
Alzheimer's disease and other dementias	4.426	0.245	8.189	1.070	4.428	4.466
Asthma	3.081	1.652	0.251	7.381	0.436	2.421
Breast cancer	3.335	0.104	3.280	0.662	4.354	7.572
Cervical cancer	0.521	0.008	0.464	0.027	0.754	0.923
Chronic obstructive pulmonary disease	10.923	0.512	10.652	4.555	9.701	1.171
Cirrhosis and other chronic liver diseases	4.110	0.040	3.730	10.459	6.151	2.818
Colon and rectum cancer	3.850	0.085	4.727	0.301	5.419	2.888
Diabetes mellitus	9.528	0.634	4.702	8.048	5.391	10.380
HIV/AIDS	1.265	0.022	0.615	0.360	1.461	30.614
Idiopathic epilepsy	0.927	0.057	0.129	0.324	0.294	1.330
Ischemic heart disease	20.509	0.427	32.957	2.067	29.959	4.535
Major depressive disorder	6.044	7.688	0.000	3.078	0.000	0.000
Multiple sclerosis	0.493	0.004	0.233	0.098	0.386	1.320
Oral disorders	2.153	72.741	0.000	30.088	0.000	4.932
Otitis media	0.124	4.062	0.002	0.652	0.003	0.189
Ovarian cancer	1.009	0.011	1.166	0.026	1.473	1.211
Parkinson's disease	0.989	0.030	1.702	0.129	1.233	1.528
Peptic ulcer disease	0.244	0.082	0.297	0.113	0.292	0.298
Pneumoconiosis	0.050	0.004	0.065	0.010	0.055	0.923
Prostate cancer	1.885	0.109	2.561	0.563	2.188	3.285
Schizophrenia	2.577	0.033	0.000	0.421	0.000	2.739
Sexually transmitted infections excluding HIV	0.116	9.568	0.010	16.096	0.014	2.481
Stroke	8.474	0.188	10.691	1.648	9.086	3.344
Tracheal, bronchus, and lung cancer	9.772	0.102	12.201	0.105	14.528	1.995
Tuberculosis	0.061	0.005	0.058	9.592	0.076	1.876
Uterine cancer	0.486	0.027	0.521	0.125	0.612	0.258

Percentage data values for 2010 (all disorders).

CAUSES-2010	DALYs	Incidence	Deaths	Prevalence	YLL	Funding
Alcohol use disorders	3.088	1.560	0.800	2.011	1.731	6.493
Alzheimer's disease and other dementias	4.483	0.245	8.240	1.074	4.493	6.436
Asthma	3.121	1.653	0.252	7.408	0.442	3.490
Breast cancer	3.378	0.104	3.301	0.665	4.418	10.912
Cervical cancer	0.528	0.008	0.466	0.027	0.766	1.330
Chronic obstructive pulmonary disease	11.063	0.512	10.718	4.572	9.844	1.688
Cirrhosis and other chronic liver diseases	4.163	0.040	3.753	10.497	6.243	4.062
Colon and rectum cancer	3.899	0.085	4.756	0.302	5.499	4.162
Diabetes mellitus	9.650	0.634	4.731	8.077	5.471	14.960
Idiopathic epilepsy	0.939	0.057	0.130	0.325	0.298	1.916
Ischemic heart disease	20.771	0.427	33.161	2.074	30.403	6.536
Major depressive disorder	6.122	7.690	0.000	3.089	0.000	0.000
Multiple sclerosis	0.500	0.004	0.234	0.098	0.392	1.902
Oral disorders	2.181	72.757	0.000	30.196	0.000	7.108
Otitis media	0.126	4.063	0.002	0.654	0.003	0.272
Ovarian cancer	1.022	0.011	1.173	0.026	1.494	1.745
Parkinson's disease	1.001	0.030	1.712	0.130	1.252	2.203
Peptic ulcer disease	0.247	0.082	0.299	0.113	0.296	0.429
Pneumoconiosis	0.051	0.004	0.065	0.010	0.056	1.330
Prostate cancer	1.909	0.109	2.577	0.565	2.221	4.734
Schizophrenia	2.610	0.033	0.000	0.422	0.000	3.947
Sexually transmitted infections excluding HIV	0.117	9.570	0.010	16.154	0.015	3.576
Stroke	8.582	0.188	10.757	1.654	9.221	4.820
Tracheal, bronchus, and lung cancer	9.897	0.102	12.277	0.106	14.744	2.875
Tuberculosis	0.061	0.005	0.059	9.627	0.077	2.703
Uterine cancer	0.492	0.027	0.524	0.126	0.621	0.372

Percentage data values for 2010 (HIV/AIDS removed).

CAUSES-2010	DALYs	Incidence	Deaths	Prevalence	YLL	Funding
Alcohol use disorders	3.157	5.727	0.800	2.881	1.731	6.990
Alzheimer's disease and other dementias	4.583	0.899	8.240	1.538	4.493	6.928
Asthma	3.190	6.067	0.252	10.612	0.442	3.757
Breast cancer	3.453	0.380	3.301	0.952	4.418	11.747
Cervical cancer	0.540	0.028	0.466	0.039	0.766	1.432
Chronic obstructive pulmonary disease	11.310	1.881	10.718	6.549	9.844	1.817
Cirrhosis and other chronic liver diseases	4.256	0.145	3.753	15.037	6.243	4.373
Colon and rectum cancer	3.986	0.313	4.756	0.432	5.499	4.480
Diabetes mellitus	9.865	2.328	4.731	11.571	5.471	16.105
Idiopathic epilepsy	0.960	0.210	0.130	0.466	0.298	2.063
Ischemic heart disease	21.234	1.567	33.161	2.971	30.403	7.036
Major depressive disorder	6.258	28.226	0.000	4.425	0.000	0.000
Multiple sclerosis	0.511	0.016	0.234	0.141	0.392	2.048
Otitis media	0.129	14.914	0.002	0.938	0.003	0.293
Ovarian cancer	1.045	0.041	1.173	0.037	1.494	1.878
Parkinson's disease	1.024	0.110	1.712	0.186	1.252	2.371
Peptic ulcer disease	0.252	0.300	0.299	0.162	0.296	0.462
Pneumoconiosis	0.052	0.017	0.065	0.014	0.056	1.432
Prostate cancer	1.952	0.399	2.577	0.810	2.221	5.096
Schizophrenia	2.668	0.123	0.000	0.605	0.000	4.249
Sexually transmitted infections excluding HIV	0.120	35.127	0.010	23.143	0.015	3.849
Stroke	8.773	0.692	10.757	2.369	9.221	5.189
Tracheal, bronchus, and lung cancer	10.118	0.373	12.277	0.151	14.744	3.095
Tuberculosis	0.063	0.017	0.059	13.791	0.077	2.910
Uterine cancer	0.503	0.100	0.524	0.180	0.621	0.400

Percentage data values for 2010 (HIV/AIDS and Oral disorders removed).

CAUSES-2011	DALYs	Incidence	Deaths	Prevalence	YLL	Funding
Alcohol use disorders	1197387	3504180	12227	7661979	449262	452
Alzheimer's disease and other dementias	1745466	553556	124656	4127860	1158221	448
Asthma	1222868	3769517	3748	28802087	111998	221
Breast cancer	1298297	233605	49367	2537480	1122558	715
Cervical cancer	205648	17552	7075	103912	197251	119
Chronic obstructive pulmonary disease	4314253	1158257	163037	17563220	2552108	108
Cirrhosis and other chronic liver diseases	1639980	87619	57598	40571770	1626960	303
Colon and rectum cancer	1515253	193628	71517	1159377	1413468	313
Diabetes mellitus	3720098	1435816	68571	31333766	1353180	1076
HIV/AIDS	459905	50150	8428	1396041	340611	3059
Idiopathic epilepsy	360790	128516	1982	1234341	76846	152
Ischemic heart disease	7972673	944768	493792	7895608	7715098	437
Major depressive disorder	2332339	17031847	0	11643867	0	
Multiple sclerosis	193248	9961	3534	375688	100256	122
Oral disorders	866371	162864636	0	116631856	0	501
Otitis media	48443	9026681	25	2483286	811	15
Ovarian cancer	391297	24759	17445	97740	378380	138
Parkinson's disease	392971	71120	26123	499249	325397	151
Peptic ulcer disease	95286	185305	4470	434772	75476	18
Pneumoconiosis	19202	10052	964	36210	13907	117
Prostate cancer	738365	246178	38505	2181637	566698	284
Schizophrenia	1000582	74256	0	1601794	0	264
Sexually transmitted infections excluding HIV	45049	21323718	157	61195726	3747	259
Stroke	3312173	420485	161664	6298071	2355049	317
Tracheal, bronchus, and lung cancer	3799183	228708	183292	405109	3742841	221
Tuberculosis	23172	9757	862	36437521	19275	209
Uterine cancer	197100	63949	8087	503773	164177	40

Raw data values for 2011 (all disorders).

CAUSES-2011	DALYs	Incidence	Deaths	Prevalence	YLL	Funding
Alcohol use disorders	3.062	1.567	0.811	1.989	1.737	4.493
Alzheimer's disease and other dementias	4.463	0.247	8.271	1.072	4.478	4.454
Asthma	3.127	1.685	0.249	7.477	0.433	2.197
Breast cancer	3.320	0.104	3.276	0.659	4.340	7.108
Cervical cancer	0.526	0.008	0.469	0.027	0.763	1.183
Chronic obstructive pulmonary disease	11.032	0.518	10.818	4.559	9.868	1.074
Cirrhosis and other chronic liver diseases	4.194	0.039	3.822	10.532	6.291	3.012
Colon and rectum cancer	3.875	0.087	4.745	0.301	5.465	3.112
Diabetes mellitus	9.513	0.642	4.550	8.134	5.232	10.697
HIV/AIDS	1.176	0.022	0.559	0.362	1.317	30.411
Idiopathic epilepsy	0.923	0.057	0.131	0.320	0.297	1.511
Ischemic heart disease	20.387	0.422	32.764	2.050	29.830	4.344
Major depressive disorder	5.964	7.615	0.000	3.023	0.000	0.000
Multiple sclerosis	0.494	0.004	0.235	0.098	0.388	1.213
Oral disorders	2.215	72.815	0.000	30.277	0.000	4.981
Otitis media	0.124	4.036	0.002	0.645	0.003	0.149
Ovarian cancer	1.001	0.011	1.158	0.025	1.463	1.372
Parkinson's disease	1.005	0.032	1.733	0.130	1.258	1.501
Peptic ulcer disease	0.244	0.083	0.297	0.113	0.292	0.179
Pneumoconiosis	0.049	0.004	0.064	0.009	0.054	1.163
Prostate cancer	1.888	0.110	2.555	0.566	2.191	2.823
Schizophrenia	2.559	0.033	0.000	0.416	0.000	2.625
Sexually transmitted infections excluding HIV	0.115	9.534	0.010	15.886	0.014	2.575
Stroke	8.469	0.188	10.727	1.635	9.106	3.151
Tracheal, bronchus, and lung cancer	9.715	0.102	12.162	0.105	14.471	2.197
Tuberculosis	0.059	0.004	0.057	9.459	0.075	2.078
Uterine cancer	0.504	0.029	0.537	0.131	0.635	0.398

Percentage data values for 2011 (all disorders).

CAUSES-2011	DALYs	Incidence	Deaths	Prevalence	YLL	Funding
Alcohol use disorders	3.098	1.567	0.816	1.996	1.760	6.457
Alzheimer's disease and other dementias	4.516	0.248	8.318	1.075	4.538	6.400
Asthma	3.164	1.686	0.250	7.504	0.439	3.157
Breast cancer	3.359	0.104	3.294	0.661	4.398	10.214
Cervical cancer	0.532	0.008	0.472	0.027	0.773	1.700
Chronic obstructive pulmonary disease	11.163	0.518	10.879	4.576	9.999	1.543
Cirrhosis and other chronic liver diseases	4.243	0.039	3.843	10.571	6.374	4.329
Colon and rectum cancer	3.921	0.087	4.772	0.302	5.538	4.471
Diabetes mellitus	9.626	0.642	4.575	8.164	5.302	15.371
Idiopathic epilepsy	0.934	0.057	0.132	0.322	0.301	2.171
Ischemic heart disease	20.629	0.422	32.948	2.057	30.228	6.243
Major depressive disorder	6.035	7.616	0.000	3.034	0.000	0.000
Multiple sclerosis	0.500	0.004	0.236	0.098	0.393	1.743
Oral disorders	2.242	72.831	0.000	30.387	0.000	7.157
Otitis media	0.125	4.037	0.002	0.647	0.003	0.214
Ovarian cancer	1.012	0.011	1.164	0.025	1.483	1.971
Parkinson's disease	1.017	0.032	1.743	0.130	1.275	2.157
Peptic ulcer disease	0.247	0.083	0.298	0.113	0.296	0.257
Pneumoconiosis	0.050	0.004	0.064	0.009	0.054	1.671
Prostate cancer	1.911	0.110	2.569	0.568	2.220	4.057
Schizophrenia	2.589	0.033	0.000	0.417	0.000	3.771
Sexually transmitted infections excluding HIV	0.117	9.536	0.011	15.944	0.015	3.700
Stroke	8.570	0.188	10.787	1.641	9.227	4.529
Tracheal, bronchus, and lung cancer	9.830	0.102	12.230	0.106	14.665	3.157
Tuberculosis	0.060	0.004	0.058	9.493	0.076	2.986
Uterine cancer	0.510	0.029	0.540	0.131	0.643	0.571

Percentage data values for 2011 (HIV/AIDS removed).

CAUSES-2011	DALYs	Incidence	Deaths	Prevalence	YLL	Funding
Alcohol use disorders	3.169	5.768	0.816	2.868	1.760	6.955
Alzheimer's disease and other dementias	4.620	0.911	8.318	1.545	4.538	6.893
Asthma	3.237	6.205	0.250	10.780	0.439	3.401
Breast cancer	3.436	0.385	3.294	0.950	4.398	11.002
Cervical cancer	0.544	0.029	0.472	0.039	0.773	1.831
Chronic obstructive pulmonary disease	11.419	1.906	10.879	6.573	9.999	1.662
Cirrhosis and other chronic liver diseases	4.341	0.144	3.843	15.185	6.374	4.662
Colon and rectum cancer	4.011	0.319	4.772	0.434	5.538	4.816
Diabetes mellitus	9.846	2.363	4.575	11.727	5.302	16.556
Idiopathic epilepsy	0.955	0.212	0.132	0.462	0.301	2.339
Ischemic heart disease	21.102	1.555	32.948	2.955	30.228	6.724
Major depressive disorder	6.173	28.034	0.000	4.358	0.000	0.000
Multiple sclerosis	0.511	0.016	0.236	0.141	0.393	1.877
Otitis media	0.128	14.858	0.002	0.929	0.003	0.231
Ovarian cancer	1.036	0.041	1.164	0.037	1.483	2.123
Parkinson's disease	1.040	0.117	1.743	0.187	1.275	2.323
Peptic ulcer disease	0.252	0.305	0.298	0.163	0.296	0.277
Pneumoconiosis	0.051	0.017	0.064	0.014	0.054	1.800
Prostate cancer	1.954	0.405	2.569	0.817	2.220	4.370
Schizophrenia	2.648	0.122	0.000	0.600	0.000	4.062
Sexually transmitted infections excluding HIV	0.119	35.099	0.011	22.904	0.015	3.985
Stroke	8.767	0.692	10.787	2.357	9.227	4.878
Tracheal, bronchus, and lung cancer	10.056	0.376	12.230	0.152	14.665	3.401
Tuberculosis	0.061	0.016	0.058	13.638	0.076	3.216
Uterine cancer	0.522	0.105	0.540	0.189	0.643	0.615

Percentage data values for 2011 (HIV/AIDS and Oral disorders removed).

CAUSES-2012	DALYs	Incidence	Deaths	Prevalence	YLL	Funding
Alcohol use disorders	1215750	3552605	12635	7732096	461300	455
Alzheimer's disease and other dementias	1777217	565358	127120	4207876	1179336	503
Asthma	1252766	3877612	3774	29598228	112502	229
Breast cancer	1299626	234090	49649	2563998	1122651	800
Cervical cancer	208996	17817	7224	105196	200481	112
Chronic obstructive pulmonary disease	4393809	1182158	166684	17899663	2609852	101
Cirrhosis and other chronic liver diseases	1679016	87313	59351	41682113	1665881	288
Colon and rectum cancer	1532851	195950	72250	1179304	1429567	302
Diabetes mellitus	3769137	1457894	67741	32188403	1336652	1061
HIV/AIDS	439830	51974	7973	1429824	319426	3074
Idiopathic epilepsy	362560	131871	2057	1235594	78883	156
Ischemic heart disease	7980232	947612	494253	7996036	7717248	468
Major depressive disorder	2301324	16816395	0	11498998	0	
Multiple sclerosis	195676	10043	3605	380743	101487	115
Oral disorders	931295	164809883	0	121290572	0	516
Otitis media	48793	9071261	25	2498882	808	17
Ovarian cancer	394142	24895	17625	97949	381173	147
Parkinson's disease	402834	78088	26781	511020	333699	154
Peptic ulcer disease	95996	189483	4479	443063	75807	20
Pneumoconiosis	19137	10012	960	36132	13835	115
Prostate cancer	751102	251665	39046	2234674	575761	257
Schizophrenia	1001131	74081	0	1604355	0	268
Sexually transmitted infections excluding HIV	45328	21473501	157	61541472	3734	275
Stroke	3333343	421144	163496	6292690	2375053	310
Tracheal, bronchus, and lung cancer	3802822	229496	184196	406600	3746322	233
Tuberculosis	22877	9349	858	36246713	19051	218
Uterine cancer	204622	66430	8382	523931	170449	42

Raw data values for 2012 (all disorders).

CAUSES-2012	DALYs	Incidence	Deaths	Prevalence	YLL	Funding
Alcohol use disorders	3.081	1.573	0.831	1.965	1.772	4.445
Alzheimer's disease and other dementias	4.504	0.250	8.361	1.070	4.531	4.914
Asthma	3.175	1.717	0.248	7.523	0.432	2.237
Breast cancer	3.293	0.104	3.266	0.652	4.313	7.816
Cervical cancer	0.530	0.008	0.475	0.027	0.770	1.094
Chronic obstructive pulmonary disease	11.134	0.523	10.964	4.550	10.026	0.987
Cirrhosis and other chronic liver diseases	4.255	0.039	3.904	10.595	6.400	2.814
Colon and rectum cancer	3.884	0.087	4.752	0.300	5.492	2.950
Diabetes mellitus	9.551	0.646	4.456	8.182	5.135	10.365
HIV/AIDS	1.115	0.023	0.524	0.363	1.227	30.031
Idiopathic epilepsy	0.919	0.058	0.135	0.314	0.303	1.524
Ischemic heart disease	20.222	0.420	32.510	2.032	29.646	4.572
Major depressive disorder	5.832	7.446	0.000	2.923	0.000	0.000
Multiple sclerosis	0.496	0.004	0.237	0.097	0.390	1.123
Oral disorders	2.360	72.977	0.000	30.829	0.000	5.041
Otitis media	0.124	4.017	0.002	0.635	0.003	0.166
Ovarian cancer	0.999	0.011	1.159	0.025	1.464	1.436
Parkinson's disease	1.021	0.035	1.762	0.130	1.282	1.504
Peptic ulcer disease	0.243	0.084	0.295	0.113	0.291	0.195
Pneumoconiosis	0.048	0.004	0.063	0.009	0.053	1.123
Prostate cancer	1.903	0.111	2.568	0.568	2.212	2.511
Schizophrenia	2.537	0.033	0.000	0.408	0.000	2.618
Sexually transmitted infections excluding HIV	0.115	9.508	0.010	15.642	0.014	2.687
Stroke	8.447	0.186	10.754	1.599	9.124	3.029
Tracheal, bronchus, and lung cancer	9.637	0.102	12.116	0.103	14.392	2.276
Tuberculosis	0.058	0.004	0.056	9.213	0.073	2.130
Uterine cancer	0.519	0.029	0.551	0.133	0.655	0.410

Percentage data values for 2012 (all disorders).

CAUSES-2012	DALYs	Incidence	Deaths	Prevalence	YLL	Funding
Alcohol use disorders	3.116	1.573	0.835	1.972	1.794	6.353
Alzheimer's disease and other dementias	4.554	0.250	8.406	1.073	4.587	7.023
Asthma	3.210	1.717	0.250	7.551	0.438	3.197
Breast cancer	3.330	0.104	3.283	0.654	4.366	11.170
Cervical cancer	0.536	0.008	0.478	0.027	0.780	1.564
Chronic obstructive pulmonary disease	11.260	0.524	11.022	4.566	10.151	1.410
Cirrhosis and other chronic liver diseases	4.303	0.039	3.924	10.633	6.479	4.021
Colon and rectum cancer	3.928	0.087	4.777	0.301	5.560	4.217
Diabetes mellitus	9.659	0.646	4.479	8.211	5.199	14.814
Idiopathic epilepsy	0.929	0.058	0.136	0.315	0.307	2.178
Ischemic heart disease	20.450	0.420	32.681	2.040	30.015	6.534
Major depressive disorder	5.897	7.448	0.000	2.933	0.000	0.000
Multiple sclerosis	0.501	0.004	0.238	0.097	0.395	1.606
Oral disorders	2.387	72.994	0.000	30.942	0.000	7.205
Otitis media	0.125	4.018	0.002	0.637	0.003	0.237
Ovarian cancer	1.010	0.011	1.165	0.025	1.482	2.052
Parkinson's disease	1.032	0.035	1.771	0.130	1.298	2.150
Peptic ulcer disease	0.246	0.084	0.296	0.113	0.295	0.279
Pneumoconiosis	0.049	0.004	0.063	0.009	0.054	1.606
Prostate cancer	1.925	0.111	2.582	0.570	2.239	3.588
Schizophrenia	2.566	0.033	0.000	0.409	0.000	3.742
Sexually transmitted infections excluding HIV	0.116	9.511	0.010	15.700	0.015	3.840
Stroke	8.542	0.187	10.811	1.605	9.237	4.328
Tracheal, bronchus, and lung cancer	9.745	0.102	12.179	0.104	14.571	3.253
Tuberculosis	0.059	0.004	0.057	9.247	0.074	3.044
Uterine cancer	0.524	0.029	0.554	0.134	0.663	0.586

Percentage data values for 2012 (HIV/AIDS removed).

CAUSES-2012	DALYs	Incidence	Deaths	Prevalence	YLL	Funding
Alcohol use disorders	3.192	5.826	0.835	2.856	1.794	6.846
Alzheimer's disease and other dementias	4.666	0.927	8.406	1.554	4.587	7.568
Asthma	3.289	6.359	0.250	10.934	0.438	3.446
Breast cancer	3.412	0.384	3.283	0.947	4.366	12.037
Cervical cancer	0.549	0.029	0.478	0.039	0.780	1.685
Chronic obstructive pulmonary disease	11.535	1.939	11.022	6.612	10.151	1.520
Cirrhosis and other chronic liver diseases	4.408	0.143	3.924	15.398	6.479	4.333
Colon and rectum cancer	4.024	0.321	4.777	0.436	5.560	4.544
Diabetes mellitus	9.895	2.391	4.479	11.891	5.199	15.964
Idiopathic epilepsy	0.952	0.216	0.136	0.456	0.307	2.347
Ischemic heart disease	20.950	1.554	32.681	2.954	30.015	7.042
Major depressive disorder	6.042	27.579	0.000	4.248	0.000	0.000
Multiple sclerosis	0.514	0.016	0.238	0.141	0.395	1.730
Otitis media	0.128	14.877	0.002	0.923	0.003	0.256
Ovarian cancer	1.035	0.041	1.165	0.036	1.482	2.212
Parkinson's disease	1.058	0.128	1.771	0.189	1.298	2.317
Peptic ulcer disease	0.252	0.311	0.296	0.164	0.295	0.301
Pneumoconiosis	0.050	0.016	0.063	0.013	0.054	1.730
Prostate cancer	1.972	0.413	2.582	0.825	2.239	3.867
Schizophrenia	2.628	0.121	0.000	0.593	0.000	4.033
Sexually transmitted infections excluding HIV	0.119	35.216	0.010	22.734	0.015	4.138
Stroke	8.751	0.691	10.811	2.325	9.237	4.664
Tracheal, bronchus, and lung cancer	9.983	0.376	12.179	0.150	14.571	3.506
Tuberculosis	0.060	0.015	0.057	13.390	0.074	3.280
Uterine cancer	0.537	0.109	0.554	0.194	0.663	0.632

Percentage data values for 2012 (HIV/AIDS and Oral disorders removed).

CAUSES-2013	DALYs	Incidence	Deaths	Prevalence	YLL	Funding
Alcohol use disorders	1234836	3599695	13089	7794623	475299	437
Alzheimer's disease and other dementias	1812160	578361	129640	4298072	1202230	504
Asthma	1280748	3979000	3816	30325907	113873	207
Breast cancer	1305244	234941	50064	2589973	1127046	657
Cervical cancer	213209	18151	7401	106805	204551	98
Chronic obstructive pulmonary disease	4483979	1205207	170969	18243454	2680587	102
Cirrhosis and other chronic liver diseases	1719964	86888	61199	42816876	1706691	282
Colon and rectum cancer	1554730	198447	73191	1200129	1449801	281
Diabetes mellitus	3824955	1476085	67235	33018301	1328703	1007
HIV/AIDS	426433	53541	7682	1465495	305147	2898
Idiopathic epilepsy	363837	135645	2131	1233952	81134	129
Ischemic heart disease	8038891	953193	497339	8112823	7770464	404
Major depressive disorder	2259483	16523501	0	11300520	0	
Multiple sclerosis	198070	10126	3686	385557	102824	112
Oral disorders	1008101	166759755	0	126594606	0	480
Otitis media	49174	9135731	26	2515160	828	14
Ovarian cancer	396586	25020	17788	98001	383578	133
Parkinson's disease	414617	87274	27531	524467	343669	135
Peptic ulcer disease	97405	193862	4528	451571	76822	19
Pneumoconiosis	19168	9907	962	36002	13870	113
Prostate cancer	767971	257409	39923	2289704	588684	286
Schizophrenia	999276	73698	0	1603415	0	232
Sexually transmitted infections excluding HIV	45721	21640863	159	61972861	3775	242
Stroke	3368000	421688	166058	6264887	2410302	282
Tracheal, bronchus, and lung cancer	3809636	230293	185241	406616	3753052	208
Tuberculosis	23035	8948	871	35942881	19288	240
Uterine cancer	213486	69232	8720	546532	177872	39

Raw data values for 2013 (all disorders).

CAUSES-2013	DALYs	Incidence	Deaths	Prevalence	YLL	Funding
Alcohol use disorders	3.093	1.579	0.850	1.938	1.806	4.580
Alzheimer's disease and other dementias	4.538	0.254	8.422	1.069	4.568	5.282
Asthma	3.208	1.745	0.248	7.541	0.433	2.170
Breast cancer	3.269	0.103	3.253	0.644	4.282	6.886
Cervical cancer	0.534	0.008	0.481	0.027	0.777	1.027
Chronic obstructive pulmonary disease	11.230	0.529	11.107	4.537	10.185	1.069
Cirrhosis and other chronic liver diseases	4.308	0.038	3.976	10.647	6.484	2.956
Colon and rectum cancer	3.894	0.087	4.755	0.298	5.508	2.945
Diabetes mellitus	9.579	0.648	4.368	8.211	5.048	10.554
HIV/AIDS	1.068	0.023	0.499	0.364	1.159	30.374
Idiopathic epilepsy	0.911	0.060	0.138	0.307	0.308	1.352
Ischemic heart disease	20.133	0.418	32.311	2.017	29.523	4.234
Major depressive disorder	5.659	7.248	0.000	2.810	0.000	0.000
Multiple sclerosis	0.496	0.004	0.239	0.096	0.391	1.174
Oral disorders	2.525	73.151	0.000	31.480	0.000	5.031
Otitis media	0.123	4.007	0.002	0.625	0.003	0.147
Ovarian cancer	0.993	0.011	1.156	0.024	1.457	1.394
Parkinson's disease	1.038	0.038	1.789	0.130	1.306	1.415
Peptic ulcer disease	0.244	0.085	0.294	0.112	0.292	0.199
Pneumoconiosis	0.048	0.004	0.062	0.009	0.053	1.184
Prostate cancer	1.923	0.113	2.594	0.569	2.237	2.998
Schizophrenia	2.503	0.032	0.000	0.399	0.000	2.432
Sexually transmitted infections excluding HIV	0.115	9.493	0.010	15.411	0.014	2.536
Stroke	8.435	0.185	10.788	1.558	9.158	2.956
Tracheal, bronchus, and lung cancer	9.541	0.101	12.035	0.101	14.259	2.180
Tuberculosis	0.058	0.004	0.057	8.938	0.073	2.515
Uterine cancer	0.535	0.030	0.566	0.136	0.676	0.409

Percentage data values for 2013 (all disorders).

CAUSES-2013	DALYs	Incidence	Deaths	Prevalence	YLL	Funding
Alcohol use disorders	3.126	1.579	0.855	1.945	1.827	6.578
Alzheimer's disease and other dementias	4.587	0.254	8.465	1.073	4.621	7.587
Asthma	3.242	1.746	0.249	7.569	0.438	3.116
Breast cancer	3.304	0.103	3.269	0.646	4.332	9.890
Cervical cancer	0.540	0.008	0.483	0.027	0.786	1.475
Chronic obstructive pulmonary disease	11.351	0.529	11.163	4.553	10.304	1.535
Cirrhosis and other chronic liver diseases	4.354	0.038	3.996	10.686	6.560	4.245
Colon and rectum cancer	3.936	0.087	4.779	0.300	5.573	4.230
Diabetes mellitus	9.683	0.648	4.390	8.241	5.107	15.159
Idiopathic epilepsy	0.921	0.060	0.139	0.308	0.312	1.942
Ischemic heart disease	20.350	0.418	32.473	2.025	29.869	6.082
Major depressive disorder	5.720	7.250	0.000	2.820	0.000	0.000
Multiple sclerosis	0.501	0.004	0.241	0.096	0.395	1.686
Oral disorders	2.552	73.168	0.000	31.595	0.000	7.226
Otitis media	0.124	4.008	0.002	0.628	0.003	0.211
Ovarian cancer	1.004	0.011	1.161	0.024	1.474	2.002
Parkinson's disease	1.050	0.038	1.798	0.131	1.321	2.032
Peptic ulcer disease	0.247	0.085	0.296	0.113	0.295	0.286
Pneumoconiosis	0.049	0.004	0.063	0.009	0.053	1.701
Prostate cancer	1.944	0.113	2.607	0.571	2.263	4.305
Schizophrenia	2.530	0.032	0.000	0.400	0.000	3.492
Sexually transmitted infections excluding HIV	0.116	9.495	0.010	15.467	0.015	3.643
Stroke	8.526	0.185	10.842	1.564	9.265	4.245
Tracheal, bronchus, and lung cancer	9.644	0.101	12.095	0.101	14.427	3.131
Tuberculosis	0.058	0.004	0.057	8.971	0.074	3.613
Uterine cancer	0.540	0.030	0.569	0.136	0.684	0.587

Percentage data values for 2013 (HIV/AIDS removed).

CAUSES-2013	DALYs	Incidence	Deaths	Prevalence	YLL	Funding
Alcohol use disorders	3.208	5.886	0.855	2.844	1.827	7.091
Alzheimer's disease and other dementias	4.708	0.946	8.465	1.568	4.621	8.178
Asthma	3.327	6.507	0.249	11.065	0.438	3.359
Breast cancer	3.391	0.384	3.269	0.945	4.332	10.660
Cervical cancer	0.554	0.030	0.483	0.039	0.786	1.590
Chronic obstructive pulmonary disease	11.648	1.971	11.163	6.656	10.304	1.655
Cirrhosis and other chronic liver diseases	4.468	0.142	3.996	15.622	6.560	4.576
Colon and rectum cancer	4.039	0.325	4.779	0.438	5.573	4.559
Diabetes mellitus	9.936	2.414	4.390	12.047	5.107	16.339
Idiopathic epilepsy	0.945	0.222	0.139	0.450	0.312	2.093
Ischemic heart disease	20.883	1.559	32.473	2.960	29.869	6.555
Major depressive disorder	5.870	27.020	0.000	4.123	0.000	0.000
Multiple sclerosis	0.515	0.017	0.241	0.141	0.395	1.817
Otitis media	0.128	14.939	0.002	0.918	0.003	0.227
Ovarian cancer	1.030	0.041	1.161	0.036	1.474	2.158
Parkinson's disease	1.077	0.143	1.798	0.191	1.321	2.190
Peptic ulcer disease	0.253	0.317	0.296	0.165	0.295	0.308
Pneumoconiosis	0.050	0.016	0.063	0.013	0.053	1.834
Prostate cancer	1.995	0.421	2.607	0.835	2.263	4.641
Schizophrenia	2.596	0.121	0.000	0.585	0.000	3.764
Sexually transmitted infections excluding HIV	0.119	35.388	0.010	22.611	0.015	3.927
Stroke	8.749	0.690	10.842	2.286	9.265	4.576
Tracheal, bronchus, and lung cancer	9.897	0.377	12.095	0.148	14.427	3.375
Tuberculosis	0.060	0.015	0.057	13.114	0.074	3.894
Uterine cancer	0.555	0.113	0.569	0.199	0.684	0.633

Percentage data values for 2013 (HIV/AIDS and Oral disorders removed).

CAUSES-2014	DALYs	Incidence	Deaths	Prevalence	YLL	Funding
Alcohol use disorders	1253672	3640302	13542	7849756	489538	475
Alzheimer's disease and other dementias	1841389	590041	131616	4377565	1220989	562
Asthma	1306434	4061908	3871	30970287	116102	241
Breast cancer	1320372	237215	50784	2616097	1140634	682
Cervical cancer	217833	18532	7585	108687	209006	116
Chronic obstructive pulmonary disease	4562857	1226302	174476	18544337	2744006	107
Cirrhosis and other chronic liver diseases	1762967	86590	63045	43842304	1749568	293
Colon and rectum cancer	1577357	200529	74101	1220183	1470991	271
Diabetes mellitus	3896891	1493441	67583	33796707	1340687	1011
HIV/AIDS	416116	56050	7413	1504397	292907	2978
Idiopathic epilepsy	365317	138875	2213	1231305	83646	154
Ischemic heart disease	8112533	957261	500581	8215640	7839797	421
Major depressive disorder	2223422	16273642	0	11130812	0	
Multiple sclerosis	201091	10207	3780	390220	104742	102
Oral disorders	1076925	168395208	0	131249829	0	483
Otitis media	49527	9214914	26	2531401	826	16
Ovarian cancer	400266	25239	17977	98502	387179	131
Parkinson's disease	425501	97836	28193	537070	352847	139
Peptic ulcer disease	98820	197921	4577	459541	77881	15
Pneumoconiosis	19196	9783	958	35944	13888	107
Prostate cancer	786736	262775	41062	2343620	603609	254
Schizophrenia	997645	73285	0	1602037	0	253
Sexually transmitted infections excluding HIV	46104	21802228	161	62478349	3821	263
Stroke	3418619	423146	169288	6256361	2459751	300
Tracheal, bronchus, and lung cancer	3817656	230676	186062	405049	3761095	254
Tuberculosis	23159	8589	879	35731851	19476	279
Uterine cancer	223107	72147	9084	569791	185999	57

Raw data values for 2014 (all disorders).

CAUSES-2014	DALYs	Incidence	Deaths	Prevalence	YLL	Funding
Alcohol use disorders	3.100	1.584	0.869	1.914	1.836	4.767
Alzheimer's disease and other dementias	4.553	0.257	8.443	1.067	4.578	5.640
Asthma	3.230	1.768	0.248	7.552	0.435	2.419
Breast cancer	3.265	0.103	3.258	0.638	4.277	6.845
Cervical cancer	0.539	0.008	0.487	0.027	0.784	1.164
Chronic obstructive pulmonary disease	11.283	0.534	11.193	4.522	10.289	1.074
Cirrhosis and other chronic liver diseases	4.359	0.038	4.044	10.691	6.560	2.941
Colon and rectum cancer	3.900	0.087	4.754	0.298	5.516	2.720
Diabetes mellitus	9.636	0.650	4.335	8.241	5.027	10.147
HIV/AIDS	1.029	0.024	0.476	0.367	1.098	29.888
Idiopathic epilepsy	0.903	0.060	0.142	0.300	0.314	1.546
Ischemic heart disease	20.060	0.417	32.112	2.003	29.397	4.225
Major depressive disorder	5.498	7.082	0.000	2.714	0.000	0.000
Multiple sclerosis	0.497	0.004	0.243	0.095	0.393	1.024
Oral disorders	2.663	73.278	0.000	32.005	0.000	4.847
Otitis media	0.122	4.010	0.002	0.617	0.003	0.161
Ovarian cancer	0.990	0.011	1.153	0.024	1.452	1.315
Parkinson's disease	1.052	0.043	1.809	0.131	1.323	1.395
Peptic ulcer disease	0.244	0.086	0.294	0.112	0.292	0.151
Pneumoconiosis	0.047	0.004	0.061	0.009	0.052	1.074
Prostate cancer	1.945	0.114	2.634	0.571	2.263	2.549
Schizophrenia	2.467	0.032	0.000	0.391	0.000	2.539
Sexually transmitted infections excluding HIV	0.114	9.487	0.010	15.235	0.014	2.640
Stroke	8.453	0.184	10.860	1.526	9.223	3.011
Tracheal, bronchus, and lung cancer	9.440	0.100	11.936	0.099	14.103	2.549
Tuberculosis	0.057	0.004	0.056	8.713	0.073	2.800
Uterine cancer	0.552	0.031	0.583	0.139	0.697	0.572

Percentage data values for 2014 (all disorders).

CAUSES-2014	DALYs	Incidence	Deaths	Prevalence	YLL	Funding
Alcohol use disorders	3.132	1.584	0.873	1.921	1.856	6.799
Alzheimer's disease and other dementias	4.601	0.257	8.483	1.071	4.629	8.045
Asthma	3.264	1.768	0.249	7.580	0.440	3.450
Breast cancer	3.299	0.103	3.273	0.640	4.325	9.762
Cervical cancer	0.544	0.008	0.489	0.027	0.792	1.660
Chronic obstructive pulmonary disease	11.400	0.534	11.246	4.539	10.403	1.532
Cirrhosis and other chronic liver diseases	4.405	0.038	4.064	10.730	6.633	4.194
Colon and rectum cancer	3.941	0.087	4.776	0.299	5.577	3.879
Diabetes mellitus	9.736	0.650	4.356	8.271	5.083	14.472
Idiopathic epilepsy	0.913	0.060	0.143	0.301	0.317	2.204
Ischemic heart disease	20.268	0.417	32.266	2.011	29.723	6.026
Major depressive disorder	5.555	7.083	0.000	2.724	0.000	0.000
Multiple sclerosis	0.502	0.004	0.244	0.096	0.397	1.460
Oral disorders	2.691	73.295	0.000	32.122	0.000	6.914
Otitis media	0.124	4.011	0.002	0.620	0.003	0.229
Ovarian cancer	1.000	0.011	1.159	0.024	1.468	1.875
Parkinson's disease	1.063	0.043	1.817	0.131	1.338	1.990
Peptic ulcer disease	0.247	0.086	0.295	0.112	0.295	0.215
Pneumoconiosis	0.048	0.004	0.062	0.009	0.053	1.532
Prostate cancer	1.966	0.114	2.647	0.574	2.288	3.636
Schizophrenia	2.493	0.032	0.000	0.392	0.000	3.622
Sexually transmitted infections excluding HIV	0.115	9.490	0.010	15.291	0.014	3.765
Stroke	8.541	0.184	10.912	1.531	9.326	4.294
Tracheal, bronchus, and lung cancer	9.538	0.100	11.993	0.099	14.259	3.636
Tuberculosis	0.058	0.004	0.057	8.745	0.074	3.994
Uterine cancer	0.557	0.031	0.585	0.139	0.705	0.816

Percentage data values for 2014 (HIV/AIDS removed).

CAUSES-2014	DALYs	Incidence	Deaths	Prevalence	YLL	Funding
Alcohol use disorders	3.219	5.933	0.873	2.830	1.856	7.304
Alzheimer's disease and other dementias	4.728	0.962	8.483	1.578	4.629	8.642
Asthma	3.354	6.621	0.249	11.167	0.440	3.706
Breast cancer	3.390	0.387	3.273	0.943	4.325	10.487
Cervical cancer	0.559	0.030	0.489	0.039	0.792	1.784
Chronic obstructive pulmonary disease	11.715	1.999	11.246	6.686	10.403	1.645
Cirrhosis and other chronic liver diseases	4.526	0.141	4.064	15.808	6.633	4.506
Colon and rectum cancer	4.050	0.327	4.776	0.440	5.577	4.167
Diabetes mellitus	10.005	2.434	4.356	12.186	5.083	15.547
Idiopathic epilepsy	0.938	0.226	0.143	0.444	0.317	2.368
Ischemic heart disease	20.829	1.560	32.266	2.962	29.723	6.474
Major depressive disorder	5.709	26.524	0.000	4.013	0.000	0.000
Multiple sclerosis	0.516	0.017	0.244	0.141	0.397	1.569
Otitis media	0.127	15.019	0.002	0.913	0.003	0.246
Ovarian cancer	1.028	0.041	1.159	0.036	1.468	2.014
Parkinson's disease	1.092	0.159	1.817	0.194	1.338	2.137
Peptic ulcer disease	0.254	0.323	0.295	0.166	0.295	0.231
Pneumoconiosis	0.049	0.016	0.062	0.013	0.053	1.645
Prostate cancer	2.020	0.428	2.647	0.845	2.288	3.906
Schizophrenia	2.561	0.119	0.000	0.578	0.000	3.891
Sexually transmitted infections excluding HIV	0.118	35.535	0.010	22.527	0.014	4.044
Stroke	8.777	0.690	10.912	2.256	9.326	4.613
Tracheal, bronchus, and lung cancer	9.802	0.376	11.993	0.146	14.259	3.906
Tuberculosis	0.059	0.014	0.057	12.884	0.074	4.290
Uterine cancer	0.573	0.118	0.585	0.205	0.705	0.877

Percentage data values for 2014 (HIV/AIDS and Oral disorders removed).

CAUSES-2015	DALYs	Incidence	Deaths	Prevelance	YLL	Funding
Alcohol use disorders	1271045	3664905	14005	7890105	503818	473
Alzheimer's disease and other dementias	1879747	604087	134411	4474882	1246205	589
Asthma	1329178	4113375	3933	31532239	118533	281
Breast cancer	1337413	240065	51610	2648263	1155785	674
Cervical cancer	222122	18885	7754	110552	213140	99
Chronic obstructive pulmonary disease	4664028	1248005	179295	18862703	2824536	97
Cirrhosis and other chronic liver diseases	1806093	86552	64842	44659748	1792530	295
Colon and rectum cancer	1612474	204662	75668	1244383	1503982	309
Diabetes mellitus	3986779	1510526	68860	34570931	1370193	1010
HIV/AIDS	402837	60369	7089	1546689	277130	3000
Idiopathic epilepsy	367784	140558	2306	1231134	86577	138
Ischemic heart disease	8247787	960980	508534	8322778	7971108	426
Major depressive disorder	2208957	16181612	0	11067813	0	95
Multiple sclerosis	204018	10269	3874	394695	106658	94
Oral disorders	1118456	169386571	0	134031181	0	493
Otitis media	49853	9280381	27	2544586	881	15
Ovarian cancer	395592	24896	17810	96776	382695	118
Parkinson's disease	438898	109640	29081	551312	364316	146
Peptic ulcer disease	100568	201935	4645	468099	79240	16
Pneumoconiosis	19480	9775	969	36424	14098	112
Prostate cancer	808997	270113	42207	2409031	620889	288
Schizophrenia	996248	72912	0	1601809	0	241
Sexually transmitted infections excluding HIV	46414	21911946	162	63040781	3852	250
Stroke	3495313	429212	173858	6330122	2524219	288
Tracheal, bronchus, and lung cancer	3817664	230610	186439	403779	3761204	349
Tuberculosis	23623	8321	900	35809429	19963	272
Uterine cancer	232330	74955	9445	592194	193802	52

Raw data values for 2015 (all disorders).

CAUSES-2015	DALYs	Incidence	Deaths	Prevalence	YLL	Funding
Alcohol use disorders	3.094	1.586	0.882	1.895	1.857	4.628
Alzheimer's disease and other dementias	4.575	0.261	8.466	1.074	4.593	5.763
Asthma	3.235	1.780	0.248	7.571	0.437	2.750
Breast cancer	3.255	0.104	3.251	0.636	4.259	6.595
Cervical cancer	0.541	0.008	0.488	0.027	0.785	0.969
Chronic obstructive pulmonary disease	11.353	0.540	11.293	4.529	10.409	0.949
Cirrhosis and other chronic liver diseases	4.396	0.037	4.084	10.723	6.606	2.886
Colon and rectum cancer	3.925	0.089	4.766	0.299	5.543	3.023
Diabetes mellitus	9.704	0.654	4.337	8.301	5.049	9.883
HIV/AIDS	0.981	0.026	0.446	0.371	1.021	29.354
Idiopathic epilepsy	0.895	0.061	0.145	0.296	0.319	1.350
Ischemic heart disease	20.076	0.416	32.029	1.998	29.375	4.168
Major depressive disorder	5.377	7.003	0.000	2.658	0.000	0.930
Multiple sclerosis	0.497	0.004	0.244	0.095	0.393	0.920
Oral disorders	2.722	73.310	0.000	32.182	0.000	4.824
Otitis media	0.121	4.017	0.002	0.611	0.003	0.147
Ovarian cancer	0.963	0.011	1.122	0.023	1.410	1.155
Parkinson's disease	1.068	0.047	1.832	0.132	1.343	1.429
Peptic ulcer disease	0.245	0.087	0.293	0.112	0.292	0.157
Pneumoconiosis	0.047	0.004	0.061	0.009	0.052	1.096
Prostate cancer	1.969	0.117	2.658	0.578	2.288	2.818
Schizophrenia	2.425	0.032	0.000	0.385	0.000	2.358
Sexually transmitted infections excluding HIV	0.113	9.483	0.010	15.137	0.014	2.446
Stroke	8.508	0.186	10.950	1.520	9.302	2.818
Tracheal, bronchus, and lung cancer	9.292	0.100	11.743	0.097	13.861	3.415
Tuberculosis	0.057	0.004	0.057	8.598	0.074	2.661
Uterine cancer	0.566	0.032	0.595	0.142	0.714	0.509

Percentage data values for 2015 (all disorders).

CAUSES-2015	DALYs	Incidence	Deaths	Prevalence	YLL	Funding
Alcohol use disorders	3.124	1.587	0.886	1.902	1.876	6.551
Alzheimer's disease and other dementias	4.621	0.262	8.504	1.078	4.640	8.158
Asthma	3.267	1.781	0.249	7.599	0.441	3.892
Breast cancer	3.288	0.104	3.265	0.638	4.303	9.335
Cervical cancer	0.546	0.008	0.491	0.027	0.794	1.371
Chronic obstructive pulmonary disease	11.465	0.540	11.343	4.546	10.516	1.343
Cirrhosis and other chronic liver diseases	4.440	0.037	4.102	10.763	6.674	4.086
Colon and rectum cancer	3.964	0.089	4.787	0.300	5.600	4.280
Diabetes mellitus	9.800	0.654	4.356	8.332	5.102	13.989
Idiopathic epilepsy	0.904	0.061	0.146	0.297	0.322	1.911
Ischemic heart disease	20.274	0.416	32.173	2.006	29.678	5.900
Major depressive disorder	5.430	7.005	0.000	2.667	0.000	1.316
Multiple sclerosis	0.502	0.004	0.245	0.095	0.397	1.302
Oral disorders	2.749	73.329	0.000	32.302	0.000	6.828
Otitis media	0.123	4.018	0.002	0.613	0.003	0.208
Ovarian cancer	0.972	0.011	1.127	0.023	1.425	1.634
Parkinson's disease	1.079	0.047	1.840	0.133	1.356	2.022
Peptic ulcer disease	0.247	0.087	0.294	0.113	0.295	0.222
Pneumoconiosis	0.048	0.004	0.061	0.009	0.052	1.551
Prostate cancer	1.989	0.117	2.670	0.581	2.312	3.989
Schizophrenia	2.449	0.032	0.000	0.386	0.000	3.338
Sexually transmitted infections excluding HIV	0.114	9.486	0.010	15.193	0.014	3.463
Stroke	8.592	0.186	10.999	1.526	9.398	3.989
Tracheal, bronchus, and lung cancer	9.384	0.100	11.795	0.097	14.004	4.834
Tuberculosis	0.058	0.004	0.057	8.630	0.074	3.767
Uterine cancer	0.571	0.032	0.598	0.143	0.722	0.720

Percentage data values for 2015 (HIV/AIDS removed).

CAUSES-2015	DALYs	Incidence	Deaths	Prevalence	YLL	Funding
Alcohol use disorders	3.213	5.949	0.886	2.809	1.876	7.031
Alzheimer's disease and other dementias	4.751	0.981	8.504	1.593	4.640	8.756
Asthma	3.360	6.677	0.249	11.226	0.441	4.177
Breast cancer	3.381	0.390	3.265	0.943	4.303	10.019
Cervical cancer	0.561	0.031	0.491	0.039	0.794	1.472
Chronic obstructive pulmonary disease	11.789	2.026	11.343	6.715	10.516	1.442
Cirrhosis and other chronic liver diseases	4.565	0.140	4.102	15.899	6.674	4.385
Colon and rectum cancer	4.076	0.332	4.787	0.443	5.600	4.593
Diabetes mellitus	10.077	2.452	4.356	12.307	5.102	15.014
Idiopathic epilepsy	0.930	0.228	0.146	0.438	0.322	2.051
Ischemic heart disease	20.848	1.560	32.173	2.963	29.678	6.333
Major depressive disorder	5.583	26.265	0.000	3.940	0.000	1.412
Multiple sclerosis	0.516	0.017	0.245	0.141	0.397	1.397
Otitis media	0.126	15.063	0.002	0.906	0.003	0.223
Ovarian cancer	1.000	0.040	1.127	0.034	1.425	1.754
Parkinson's disease	1.109	0.178	1.840	0.196	1.356	2.170
Peptic ulcer disease	0.254	0.328	0.294	0.167	0.295	0.238
Pneumoconiosis	0.049	0.016	0.061	0.013	0.052	1.665
Prostate cancer	2.045	0.438	2.670	0.858	2.312	4.281
Schizophrenia	2.518	0.118	0.000	0.570	0.000	3.583
Sexually transmitted infections excluding HIV	0.117	35.566	0.010	22.443	0.014	3.716
Stroke	8.835	0.697	10.999	2.254	9.398	4.281
Tracheal, bronchus, and lung cancer	9.650	0.374	11.795	0.144	14.004	5.188
Tuberculosis	0.060	0.014	0.057	12.748	0.074	4.043
Uterine cancer	0.587	0.122	0.598	0.211	0.722	0.773

Percentage data values for 2015 (HIV/AIDS and Oral disorders removed).

CAUSES-2016	DALYs	Incidence	Deaths	Prevelance	YLL	Funding
Alcohol use disorders	1281471	3660464	14347	7912711	513195	486
Alzheimer's disease and other dementias	1912733	617516	136673	4567579	1267029	929
Asthma	1352572	4085978	3993	32094255	121526	266
Breast cancer	1357252	245288	52338	2682108	1173571	656
Cervical cancer	225152	19255	7859	112767	215994	99
Chronic obstructive pulmonary disease	4731156	1256867	182728	19012133	2893967	97
Cirrhosis and other chronic liver diseases	1844814	88604	66395	45300531	1831037	293
Colon and rectum cancer	1658393	211415	77499	1274702	1546764	274
Diabetes mellitus	4075316	1522441	70534	35195617	1411037	1084
HIV/AIDS	421683	63958	7430	1593857	295299	3000
Idiopathic epilepsy	368253	137719	2366	1226099	88742	153
Ischemic heart disease	8409870	954315	516143	8371401	8132665	419
Major depressive disorder	2214670	16244008	0	11108361	0	103
Multiple sclerosis	206648	10338	3952	399101	108317	97
Oral disorders	1134303	169593939	0	135126174	0	518
Otitis media	49903	9272078	27	2548358	881	16
Ovarian cancer	401393	25321	18076	98206	388285	144
Parkinson's disease	450517	129205	29748	564899	374123	161
Peptic ulcer disease	103033	204647	4731	477516	81304	9
Pneumoconiosis	20211	9905	996	38253	14579	127
Prostate cancer	834169	280228	43456	2484118	639873	253
Schizophrenia	989240	72172	0	1592399	0	254
Sexually transmitted infections excluding HIV	46720	21925923	165	63583161	3940	260
Stroke	3606504	442397	177437	6667884	2590160	308
Tracheal, bronchus, and lung cancer	3860621	234041	188448	410464	3803348	331
Tuberculosis	24342	8071	927	36117608	20664	290
Uterine cancer	242076	78451	9788	620242	201740	50

Raw data values for 2016 (all disorders).

CAUSES-2016	DALYs	Incidence	Deaths	Prevalence	YLL	Funding
Alcohol use disorders	3.064	1.582	0.888	1.879	1.851	4.552
Alzheimer's disease and other dementias	4.573	0.267	8.457	1.084	4.571	8.701
Asthma	3.234	1.766	0.247	7.620	0.438	2.491
Breast cancer	3.245	0.106	3.239	0.637	4.234	6.144
Cervical cancer	0.538	0.008	0.486	0.027	0.779	0.927
Chronic obstructive pulmonary disease	11.312	0.543	11.307	4.514	10.441	0.908
Cirrhosis and other chronic liver diseases	4.411	0.038	4.108	10.756	6.606	2.744
Colon and rectum cancer	3.965	0.091	4.796	0.303	5.580	2.566
Diabetes mellitus	9.744	0.658	4.365	8.356	5.091	10.153
HIV/AIDS	1.008	0.028	0.460	0.378	1.065	28.098
Idiopathic epilepsy	0.881	0.060	0.146	0.291	0.320	1.433
Ischemic heart disease	20.108	0.412	31.938	1.988	29.341	3.924
Major depressive disorder	5.295	7.020	0.000	2.637	0.000	0.965
Multiple sclerosis	0.494	0.004	0.245	0.095	0.391	0.908
Oral disorders	2.712	73.292	0.000	32.083	0.000	4.852
Otitis media	0.119	4.007	0.002	0.605	0.003	0.150
Ovarian cancer	0.960	0.011	1.119	0.023	1.401	1.349
Parkinson's disease	1.077	0.056	1.841	0.134	1.350	1.508
Peptic ulcer disease	0.246	0.088	0.293	0.113	0.293	0.084
Pneumoconiosis	0.048	0.004	0.062	0.009	0.053	1.189
Prostate cancer	1.995	0.121	2.689	0.590	2.309	2.370
Schizophrenia	2.365	0.031	0.000	0.378	0.000	2.379
Sexually transmitted infections excluding HIV	0.112	9.476	0.010	15.096	0.014	2.435
Stroke	8.623	0.191	10.980	1.583	9.345	2.885
Tracheal, bronchus, and lung cancer	9.231	0.101	11.661	0.097	13.722	3.100
Tuberculosis	0.058	0.003	0.057	8.575	0.075	2.716
Uterine cancer	0.579	0.034	0.606	0.147	0.728	0.468

Percentage data values for 2016 (all disorders).

CAUSES-2016	DALYs	Incidence	Deaths	Prevalence	YLL	Funding
Alcohol use disorders	3.095	1.582	0.892	1.886	1.871	6.331
Alzheimer's disease and other dementias	4.620	0.267	8.496	1.089	4.620	12.101
Asthma	3.267	1.766	0.248	7.649	0.443	3.465
Breast cancer	3.278	0.106	3.254	0.639	4.280	8.545
Cervical cancer	0.544	0.008	0.489	0.027	0.788	1.290
Chronic obstructive pulmonary disease	11.428	0.543	11.359	4.531	10.553	1.264
Cirrhosis and other chronic liver diseases	4.456	0.038	4.127	10.796	6.677	3.817
Colon and rectum cancer	4.006	0.091	4.818	0.304	5.640	3.569
Diabetes mellitus	9.843	0.658	4.385	8.388	5.145	14.120
Idiopathic epilepsy	0.889	0.060	0.147	0.292	0.324	1.993
Ischemic heart disease	20.313	0.413	32.086	1.995	29.657	5.458
Major depressive disorder	5.349	7.022	0.000	2.647	0.000	1.342
Multiple sclerosis	0.499	0.004	0.246	0.095	0.395	1.264
Oral disorders	2.740	73.312	0.000	32.205	0.000	6.747
Otitis media	0.121	4.008	0.002	0.607	0.003	0.208
Ovarian cancer	0.970	0.011	1.124	0.023	1.416	1.876
Parkinson's disease	1.088	0.056	1.849	0.135	1.364	2.097
Peptic ulcer disease	0.249	0.088	0.294	0.114	0.296	0.117
Pneumoconiosis	0.049	0.004	0.062	0.009	0.053	1.654
Prostate cancer	2.015	0.121	2.701	0.592	2.333	3.296
Schizophrenia	2.389	0.031	0.000	0.380	0.000	3.309
Sexually transmitted infections excluding HIV	0.113	9.478	0.010	15.154	0.014	3.387
Stroke	8.711	0.191	11.030	1.589	9.445	4.012
Tracheal, bronchus, and lung cancer	9.325	0.101	11.715	0.098	13.869	4.312
Tuberculosis	0.059	0.003	0.058	8.608	0.075	3.778
Uterine cancer	0.585	0.034	0.608	0.148	0.736	0.651

Percentage data values for 2016 (HIV/AIDS removed).

CAUSES-2016	DALYs	Incidence	Deaths	Prevalence	YLL	Funding
Alcohol use disorders	3.182	5.929	0.892	2.782	1.871	6.789
Alzheimer's disease and other dementias	4.750	1.000	8.496	1.606	4.620	12.977
Asthma	3.359	6.618	0.248	11.283	0.443	3.716
Breast cancer	3.371	0.397	3.254	0.943	4.280	9.163
Cervical cancer	0.559	0.031	0.489	0.040	0.788	1.383
Chronic obstructive pulmonary disease	11.749	2.036	11.359	6.684	10.553	1.355
Cirrhosis and other chronic liver diseases	4.581	0.144	4.127	15.925	6.677	4.093
Colon and rectum cancer	4.118	0.342	4.818	0.448	5.640	3.827
Diabetes mellitus	10.121	2.466	4.385	12.373	5.145	15.142
Idiopathic epilepsy	0.915	0.223	0.147	0.431	0.324	2.137
Ischemic heart disease	20.885	1.546	32.086	2.943	29.657	5.853
Major depressive disorder	5.500	26.312	0.000	3.905	0.000	1.439
Multiple sclerosis	0.513	0.017	0.246	0.140	0.395	1.355
Otitis media	0.124	15.019	0.002	0.896	0.003	0.223
Ovarian cancer	0.997	0.041	1.124	0.035	1.416	2.011
Parkinson's disease	1.119	0.209	1.849	0.199	1.364	2.249
Peptic ulcer disease	0.256	0.331	0.294	0.168	0.296	0.126
Pneumoconiosis	0.050	0.016	0.062	0.013	0.053	1.774
Prostate cancer	2.072	0.454	2.701	0.873	2.333	3.534
Schizophrenia	2.457	0.117	0.000	0.560	0.000	3.548
Sexually transmitted infections excluding HIV	0.116	35.515	0.010	22.352	0.014	3.632
Stroke	8.956	0.717	11.030	2.344	9.445	4.302
Tracheal, bronchus, and lung cancer	9.588	0.379	11.715	0.144	13.869	4.624
Tuberculosis	0.060	0.013	0.058	12.697	0.075	4.051
Uterine cancer	0.601	0.127	0.608	0.218	0.736	0.698

Percentage data values for 2016 (HIV/AIDS and Oral disorders removed).

CAUSES-2017	DALYs	Incidence	Deaths	Prevalence	YLL	Funding
Alcohol use disorders	1272479	3652762	14169	7937487	503082	500
Alzheimer's disease and other dementias	1946869	633404	138750	4679349	1286583	1361
Asthma	1371838	4048641	3995	32655407	120531	286
Breast cancer	1361456	245460	52464	2719039	1176508	689
Cervical cancer	224429	19143	7835	112031	215329	114
Chronic obstructive pulmonary disease	4765948	1268798	183776	19253345	2921230	100
Cirrhosis and other chronic liver diseases	1826308	90828	65977	45911664	1812278	285
Colon and rectum cancer	1680165	214245	78717	1306677	1566075	270
Diabetes mellitus	4156772	1533453	72239	35870723	1440894	1108
HIV/AIDS	419493	65569	7338	1642817	292216	3000
Idiopathic epilepsy	362362	133252	2324	1211656	86798	154
Ischemic heart disease	8483076	948571	521605	8467586	8203553	444
Major depressive disorder	2223399	16332108	0	11165604	0	104
Multiple sclerosis	208444	10401	3998	402880	109377	111
Oral disorders	1148157	169632669	0	136008885	0	541
Otitis media	49889	9271941	27	2548955	879	9
Ovarian cancer	409169	25705	18431	99371	395874	151
Parkinson's disease	458098	146969	30014	581401	379569	168
Peptic ulcer disease	103367	207451	4732	487553	81226	8
Pneumoconiosis	20932	10077	1021	40903	14947	114
Prostate cancer	861331	288342	44795	2564942	661129	239
Schizophrenia	983755	71551	0	1585536	0	243
Sexually transmitted infections excluding HIV	46969	21936097	166	64093683	3954	322
Stroke	3667978	457048	177920	7027598	2602667	331
Tracheal, bronchus, and lung cancer	3920138	237001	191190	415535	3862206	352
Tuberculosis	24349	7954	929	36406100	20623	347
Uterine cancer	241020	77732	9763	614300	201086	45

Raw data values for 2017 (all disorders).

CAUSES-2017	DALYs	Incidence	Deaths	Prevalence	YLL	Funding
Alcohol use disorders	3.013	1.577	0.868	1.864	1.799	4.388
Alzheimer's disease and other dementias	4.609	0.274	8.501	1.099	4.602	11.943
Asthma	3.248	1.748	0.245	7.669	0.431	2.510
Breast cancer	3.223	0.106	3.214	0.639	4.208	6.046
Cervical cancer	0.531	0.008	0.480	0.026	0.770	1.000
Chronic obstructive pulmonary disease	11.284	0.548	11.260	4.522	10.448	0.878
Cirrhosis and other chronic liver diseases	4.324	0.039	4.042	10.782	6.482	2.501
Colon and rectum cancer	3.978	0.093	4.823	0.307	5.601	2.369
Diabetes mellitus	9.841	0.662	4.426	8.424	5.154	9.723
HIV/AIDS	0.993	0.028	0.450	0.386	1.045	26.325
Idiopathic epilepsy	0.858	0.058	0.142	0.285	0.310	1.351
Ischemic heart disease	20.084	0.410	31.958	1.989	29.342	3.896
Major depressive disorder	5.264	7.053	0.000	2.622	0.000	0.913
Multiple sclerosis	0.493	0.004	0.245	0.095	0.391	0.974
Oral disorders	2.718	73.254	0.000	31.941	0.000	4.747
Otitis media	0.118	4.004	0.002	0.599	0.003	0.079
Ovarian cancer	0.969	0.011	1.129	0.023	1.416	1.325
Parkinson's disease	1.085	0.063	1.839	0.137	1.358	1.474
Peptic ulcer disease	0.245	0.090	0.290	0.114	0.291	0.070
Pneumoconiosis	0.050	0.004	0.063	0.010	0.053	1.000
Prostate cancer	2.039	0.125	2.744	0.602	2.365	2.097
Schizophrenia	2.329	0.031	0.000	0.372	0.000	2.132
Sexually transmitted infections excluding HIV	0.111	9.473	0.010	15.052	0.014	2.826
Stroke	8.684	0.197	10.901	1.650	9.309	2.905
Tracheal, bronchus, and lung cancer	9.281	0.102	11.714	0.098	13.814	3.089
Tuberculosis	0.058	0.003	0.057	8.550	0.074	3.045
Uterine cancer	0.571	0.034	0.598	0.144	0.719	0.395

Percentage data values for 2017 (all disorders).

CAUSES-2017	DALYs	Incidence	Deaths	Prevalence	YLL	Funding
Alcohol use disorders	3.043	1.578	0.872	1.871	1.818	5.955
Alzheimer's disease and other dementias	4.655	0.274	8.539	1.103	4.650	16.210
Asthma	3.280	1.749	0.246	7.699	0.436	3.406
Breast cancer	3.256	0.106	3.229	0.641	4.252	8.206
Cervical cancer	0.537	0.008	0.482	0.026	0.778	1.358
Chronic obstructive pulmonary disease	11.397	0.548	11.310	4.539	10.559	1.191
Cirrhosis and other chronic liver diseases	4.367	0.039	4.061	10.824	6.550	3.394
Colon and rectum cancer	4.018	0.093	4.845	0.308	5.661	3.216
Diabetes mellitus	9.940	0.662	4.446	8.457	5.208	13.197
Idiopathic epilepsy	0.867	0.058	0.143	0.286	0.314	1.834
Ischemic heart disease	20.285	0.410	32.102	1.996	29.652	5.288
Major depressive disorder	5.317	7.055	0.000	2.632	0.000	1.239
Multiple sclerosis	0.498	0.004	0.246	0.095	0.395	1.322
Oral disorders	2.746	73.275	0.000	32.065	0.000	6.444
Otitis media	0.119	4.005	0.002	0.601	0.003	0.107
Ovarian cancer	0.978	0.011	1.134	0.023	1.431	1.798
Parkinson's disease	1.095	0.063	1.847	0.137	1.372	2.001
Peptic ulcer disease	0.247	0.090	0.291	0.115	0.294	0.095
Pneumoconiosis	0.050	0.004	0.063	0.010	0.054	1.358
Prostate cancer	2.060	0.125	2.757	0.605	2.390	2.847
Schizophrenia	2.352	0.031	0.000	0.374	0.000	2.894
Sexually transmitted infections excluding HIV	0.112	9.476	0.010	15.110	0.014	3.835
Stroke	8.771	0.197	10.950	1.657	9.407	3.942
Tracheal, bronchus, and lung cancer	9.374	0.102	11.767	0.098	13.960	4.192
Tuberculosis	0.058	0.003	0.057	8.583	0.075	4.133
Uterine cancer	0.576	0.034	0.601	0.145	0.727	0.536

Percentage data values for 2017 (HIV/AIDS removed).

CAUSES-2017	DALYs	Incidence	Deaths	Prevalence	YLL	Funding
Alcohol use disorders	3.129	5.904	0.872	2.755	1.818	6.365
Alzheimer's disease and other dementias	4.787	1.024	8.539	1.624	4.650	17.327
Asthma	3.373	6.544	0.246	11.332	0.436	3.641
Breast cancer	3.348	0.397	3.229	0.944	4.252	8.771
Cervical cancer	0.552	0.031	0.482	0.039	0.778	1.451
Chronic obstructive pulmonary disease	11.718	2.051	11.310	6.681	10.559	1.273
Cirrhosis and other chronic liver diseases	4.490	0.147	4.061	15.933	6.550	3.628
Colon and rectum cancer	4.131	0.346	4.845	0.453	5.661	3.437
Diabetes mellitus	10.221	2.479	4.446	12.448	5.208	14.106
Idiopathic epilepsy	0.891	0.215	0.143	0.420	0.314	1.961
Ischemic heart disease	20.858	1.533	32.102	2.939	29.652	5.652
Major depressive disorder	5.467	26.398	0.000	3.875	0.000	1.324
Multiple sclerosis	0.513	0.017	0.246	0.140	0.395	1.413
Otitis media	0.123	14.986	0.002	0.885	0.003	0.115
Ovarian cancer	1.006	0.042	1.134	0.034	1.431	1.922
Parkinson's disease	1.126	0.238	1.847	0.202	1.372	2.139
Peptic ulcer disease	0.254	0.335	0.291	0.169	0.294	0.102
Pneumoconiosis	0.051	0.016	0.063	0.014	0.054	1.451
Prostate cancer	2.118	0.466	2.757	0.890	2.390	3.043
Schizophrenia	2.419	0.116	0.000	0.550	0.000	3.094
Sexually transmitted infections excluding HIV	0.115	35.456	0.010	22.242	0.014	4.099
Stroke	9.019	0.739	10.950	2.439	9.407	4.214
Tracheal, bronchus, and lung cancer	9.639	0.383	11.767	0.144	13.960	4.481
Tuberculosis	0.060	0.013	0.057	12.634	0.075	4.418
Uterine cancer	0.593	0.126	0.601	0.213	0.727	0.573

Percentage data values for 2017 (HIV/AIDS and Oral disorders removed).

CAUSES-2018	DALYs	Incidence	Deaths	Prevelance	YLL	Funding
Alcohol use disorders	1272159	3677888	14162	8010237	496915	534
Alzheimer's disease and other dementias	1991457	650971	141681	4802306	1315494	1789
Asthma	1391465	4077411	4033	33252089	119294	304
Breast cancer	1377157	248802	53344	2766891	1189450	721
Cervical cancer	224434	19145	7881	111561	215349	112
Chronic obstructive pulmonary disease	4900408	1295099	190016	19707532	3017479	111
Cirrhosis and other chronic liver diseases	1831531	91177	66816	46503906	1817505	324
Colon and rectum cancer	1722726	221028	81395	1341552	1605470	314
Diabetes mellitus	4292174	1566339	75081	37090052	1483540	1039
HIV/AIDS	417683	66790	7200	1692610	287119	2995
Idiopathic epilepsy	350052	128934	2309	1168641	84848	184
Ischemic heart disease	8749762	952607	540943	8655104	8462811	444
Major depressive disorder	2232233	16425294	0	11228315	0	107
Multiple sclerosis	210083	10438	4067	406036	110441	112
Oral disorders	1169609	170021765	0	137045525	0	575
Otitis media	49978	9454223	28	2557889	881	10
Ovarian cancer	416317	26166	18848	100449	402829	159
Parkinson's disease	477809	153147	31277	600161	396912	193
Peptic ulcer disease	105372	210024	4862	494987	82885	8
Pneumoconiosis	22400	10118	1099	43474	16055	134
Prostate cancer	898629	299901	46829	2650952	690786	261
Schizophrenia	987184	71423	0	1594121	0	248
Sexually transmitted infections excluding HIV	47271	22117694	169	64660369	3976	354
Stroke	3758985	461639	183505	7134676	2674335	348
Tracheal, bronchus, and lung cancer	4068157	246824	199320	430594	4008045	403
Tuberculosis	24735	8017	956	36655874	20932	403
Uterine cancer	243795	78419	9926	618484	203556	47

Raw data values for 2018 (all disorders).

CAUSES-2018	DALYs	Incidence	Deaths	Prevalence	YLL	Funding
Alcohol use disorders	2.943	1.581	0.840	1.857	1.731	4.365
Alzheimer's disease and other dementias	4.606	0.280	8.405	1.113	4.583	14.624
Asthma	3.218	1.753	0.239	7.709	0.416	2.485
Breast cancer	3.185	0.107	3.164	0.641	4.143	5.894
Cervical cancer	0.519	0.008	0.468	0.026	0.750	0.916
Chronic obstructive pulmonary disease	11.335	0.557	11.272	4.569	10.511	0.907
Cirrhosis and other chronic liver diseases	4.236	0.039	3.964	10.782	6.331	2.649
Colon and rectum cancer	3.985	0.095	4.828	0.311	5.593	2.567
Diabetes mellitus	9.928	0.673	4.454	8.599	5.168	8.493
HIV/AIDS	0.966	0.029	0.427	0.392	1.000	24.483
Idiopathic epilepsy	0.810	0.055	0.137	0.271	0.296	1.504
Ischemic heart disease	20.238	0.410	32.089	2.007	29.480	3.630
Major depressive disorder	5.163	7.062	0.000	2.603	0.000	0.875
Multiple sclerosis	0.486	0.004	0.241	0.094	0.385	0.916
Oral disorders	2.705	73.099	0.000	31.773	0.000	4.700
Otitis media	0.116	4.065	0.002	0.593	0.003	0.082
Ovarian cancer	0.963	0.011	1.118	0.023	1.403	1.300
Parkinson's disease	1.105	0.066	1.855	0.139	1.383	1.578
Peptic ulcer disease	0.244	0.090	0.288	0.115	0.289	0.065
Pneumoconiosis	0.052	0.004	0.065	0.010	0.056	1.095
Prostate cancer	2.079	0.129	2.778	0.615	2.406	2.134
Schizophrenia	2.283	0.031	0.000	0.370	0.000	2.027
Sexually transmitted infections excluding HIV	0.109	9.509	0.010	14.991	0.014	2.894
Stroke	8.695	0.198	10.886	1.654	9.316	2.845
Tracheal, bronchus, and lung cancer	9.410	0.106	11.824	0.100	13.962	3.294
Tuberculosis	0.057	0.003	0.057	8.498	0.073	3.294
Uterine cancer	0.564	0.034	0.589	0.143	0.709	0.384

Percentage data values for 2018 (all disorders).

CAUSES-2018	DALYs	Incidence	Deaths	Prevalence	YLL	Funding
Alcohol use disorders	2.971	1.582	0.844	1.864	1.748	5.780
Alzheimer's disease and other dementias	4.651	0.280	8.441	1.118	4.629	19.366
Asthma	3.250	1.754	0.240	7.740	0.420	3.291
Breast cancer	3.216	0.107	3.178	0.644	4.185	7.805
Cervical cancer	0.524	0.008	0.470	0.026	0.758	1.212
Chronic obstructive pulmonary disease	11.445	0.557	11.320	4.587	10.618	1.202
Cirrhosis and other chronic liver diseases	4.278	0.039	3.981	10.824	6.395	3.507
Colon and rectum cancer	4.024	0.095	4.849	0.312	5.649	3.399
Diabetes mellitus	10.025	0.674	4.473	8.633	5.220	11.247
Idiopathic epilepsy	0.818	0.055	0.138	0.272	0.299	1.992
Ischemic heart disease	20.436	0.410	32.227	2.015	29.778	4.806
Major depressive disorder	5.214	7.064	0.000	2.613	0.000	1.158
Multiple sclerosis	0.491	0.004	0.242	0.095	0.389	1.212
Oral disorders	2.732	73.120	0.000	31.898	0.000	6.224
Otitis media	0.117	4.066	0.002	0.595	0.003	0.108
Ovarian cancer	0.972	0.011	1.123	0.023	1.417	1.721
Parkinson's disease	1.116	0.066	1.863	0.140	1.397	2.089
Peptic ulcer disease	0.246	0.090	0.290	0.115	0.292	0.087
Pneumoconiosis	0.052	0.004	0.065	0.010	0.056	1.451
Prostate cancer	2.099	0.129	2.790	0.617	2.431	2.825
Schizophrenia	2.306	0.031	0.000	0.371	0.000	2.685
Sexually transmitted infections excluding HIV	0.110	9.512	0.010	15.050	0.014	3.832
Stroke	8.779	0.199	10.932	1.661	9.410	3.767
Tracheal, bronchus, and lung cancer	9.502	0.106	11.875	0.100	14.103	4.362
Tuberculosis	0.058	0.003	0.057	8.532	0.074	4.362
Uterine cancer	0.569	0.034	0.591	0.144	0.716	0.509

Percentage data values for 2018 (HIV/AIDS removed).

CAUSES-2018	DALYs	Incidence	Deaths	Prevalence	YLL	Funding
Alcohol use disorders	3.055	5.884	0.844	2.738	1.748	6.164
Alzheimer's disease and other dementias	4.782	1.042	8.441	1.641	4.629	20.651
Asthma	3.341	6.524	0.240	11.365	0.420	3.509
Breast cancer	3.307	0.398	3.178	0.946	4.185	8.323
Cervical cancer	0.539	0.031	0.470	0.038	0.758	1.293
Chronic obstructive pulmonary disease	11.767	2.072	11.320	6.736	10.618	1.281
Cirrhosis and other chronic liver diseases	4.398	0.146	3.981	15.894	6.395	3.740
Colon and rectum cancer	4.137	0.354	4.849	0.459	5.649	3.625
Diabetes mellitus	10.306	2.506	4.473	12.677	5.220	11.994
Idiopathic epilepsy	0.841	0.206	0.138	0.399	0.299	2.124
Ischemic heart disease	21.010	1.524	32.227	2.958	29.778	5.125
Major depressive disorder	5.360	26.279	0.000	3.838	0.000	1.235
Multiple sclerosis	0.504	0.017	0.242	0.139	0.389	1.293
Otitis media	0.120	15.126	0.002	0.874	0.003	0.115
Ovarian cancer	1.000	0.042	1.123	0.034	1.417	1.835
Parkinson's disease	1.147	0.245	1.863	0.205	1.397	2.228
Peptic ulcer disease	0.253	0.336	0.290	0.169	0.292	0.092
Pneumoconiosis	0.054	0.016	0.065	0.015	0.056	1.547
Prostate cancer	2.158	0.480	2.790	0.906	2.431	3.013
Schizophrenia	2.370	0.114	0.000	0.545	0.000	2.863
Sexually transmitted infections excluding HIV	0.114	35.387	0.010	22.100	0.014	4.086
Stroke	9.026	0.739	10.932	2.438	9.410	4.017
Tracheal, bronchus, and lung cancer	9.768	0.395	11.875	0.147	14.103	4.652
Tuberculosis	0.059	0.013	0.057	12.528	0.074	4.652
Uterine cancer	0.585	0.125	0.591	0.211	0.716	0.543

Percentage data values for 2018 (HIV/AIDS and Oral disorders removed).

CAUSES-2019	DALYs	Incidence	Deaths	Prevelance	YLL	Funding
Alcohol use disorders	1275587	3728190	14044	8129256	490603	556
Alzheimer's disease and other dementias	2026882	665941	143919	4902695	1339084	2240
Asthma	1414555	4143124	4072	33954468	117896	313
Breast cancer	1403392	254486	55021	2817911	1212430	709
Cervical cancer	224783	19112	7995	110458	215759	106
Chronic obstructive pulmonary disease	5021538	1322417	195825	20147917	3100425	112
Cirrhosis and other chronic liver diseases	1825800	90854	67286	47083998	1811949	351
Colon and rectum cancer	1760640	227242	84026	1376788	1640648	294
Diabetes mellitus	4461171	1622424	77719	38858416	1519395	1099
HIV/AIDS	415325	67134	7053	1743128	281414	3037
Idiopathic epilepsy	330421	122703	2299	1090179	83363	188
Ischemic heart disease	8948089	959514	557649	8867236	8651610	421
Major depressive disorder	2242301	16533459	0	11303861	0	131
Multiple sclerosis	211385	10465	4126	409217	111217	111
Oral disorders	1198547	170691941	0	138161388	0	613
Otitis media	50140	9809439	29	2573148	886	13
Ovarian cancer	426504	26786	19503	101873	412772	168
Parkinson's disease	492368	153614	32211	618190	409246	224
Peptic ulcer disease	106986	211088	4990	499172	84261	8
Pneumoconiosis	23286	10014	1133	46307	16540	146
Prostate cancer	926635	308584	48323	2731473	712794	263
Schizophrenia	993335	71349	0	1607948	0	263
Sexually transmitted infections excluding HIV	47592	22426038	173	65267526	3999	354
Stroke	3826274	455717	189456	7091313	2739238	350
Tracheal, bronchus, and lung cancer	4186491	254808	206196	444083	4124648	419
Tuberculosis	25031	8108	978	36886919	21133	488
Uterine cancer	249557	80070	10260	630526	208630	36

Raw data values for 2019 (all disorders).

CAUSES-2019	DALYs	Incidence	Deaths	Prevalence	YLL	Funding
Alcohol use disorders	2.892	1.591	0.810	1.858	1.674	4.273
Alzheimer's disease and other dementias	4.595	0.284	8.298	1.121	4.569	17.214
Asthma	3.207	1.768	0.235	7.762	0.402	2.405
Breast cancer	3.181	0.109	3.173	0.644	4.137	5.448
Cervical cancer	0.510	0.008	0.461	0.025	0.736	0.815
Chronic obstructive pulmonary disease	11.383	0.564	11.291	4.606	10.578	0.861
Cirrhosis and other chronic liver diseases	4.139	0.039	3.880	10.763	6.182	2.697
Colon and rectum cancer	3.991	0.097	4.845	0.315	5.598	2.259
Diabetes mellitus	10.113	0.693	4.481	8.883	5.184	8.445
HIV/AIDS	0.941	0.029	0.407	0.398	0.960	23.338
Idiopathic epilepsy	0.749	0.052	0.133	0.249	0.284	1.445
Ischemic heart disease	20.284	0.410	32.154	2.027	29.518	3.235
Major depressive disorder	5.083	7.057	0.000	2.584	0.000	1.007
Multiple sclerosis	0.479	0.004	0.238	0.094	0.379	0.853
Oral disorders	2.717	72.860	0.000	31.583	0.000	4.711
Otitis media	0.114	4.187	0.002	0.588	0.003	0.100
Ovarian cancer	0.967	0.011	1.125	0.023	1.408	1.291
Parkinson's disease	1.116	0.066	1.857	0.141	1.396	1.721
Peptic ulcer disease	0.243	0.090	0.288	0.114	0.287	0.061
Pneumoconiosis	0.053	0.004	0.065	0.011	0.056	1.122
Prostate cancer	2.101	0.132	2.786	0.624	2.432	2.021
Schizophrenia	2.252	0.030	0.000	0.368	0.000	2.021
Sexually transmitted infections excluding HIV	0.108	9.573	0.010	14.920	0.014	2.720
Stroke	8.673	0.195	10.924	1.621	9.346	2.690
Tracheal, bronchus, and lung cancer	9.490	0.109	11.889	0.102	14.073	3.220
Tuberculosis	0.057	0.003	0.056	8.432	0.072	3.750
Uterine cancer	0.566	0.034	0.592	0.144	0.712	0.277

Percentage data values for 2019 (all disorders).

CAUSES-2019	DALYs	Incidence	Deaths	Prevalence	YLL	Funding
Alcohol use disorders	2.919	1.592	0.813	1.866	1.690	5.573
Alzheimer's disease and other dementias	4.638	0.284	8.332	1.125	4.613	22.454
Asthma	3.237	1.769	0.236	7.793	0.406	3.138
Breast cancer	3.211	0.109	3.186	0.647	4.177	7.107
Cervical cancer	0.514	0.008	0.463	0.025	0.743	1.063
Chronic obstructive pulmonary disease	11.491	0.565	11.338	4.624	10.681	1.123
Cirrhosis and other chronic liver diseases	4.178	0.039	3.896	10.806	6.242	3.518
Colon and rectum cancer	4.029	0.097	4.865	0.316	5.652	2.947
Diabetes mellitus	10.209	0.693	4.500	8.918	5.234	11.016
Idiopathic epilepsy	0.756	0.052	0.133	0.250	0.287	1.885
Ischemic heart disease	20.477	0.410	32.286	2.035	29.804	4.220
Major depressive disorder	5.131	7.059	0.000	2.594	0.000	1.313
Multiple sclerosis	0.484	0.004	0.239	0.094	0.383	1.113
Oral disorders	2.743	72.881	0.000	31.709	0.000	6.145
Otitis media	0.115	4.188	0.002	0.591	0.003	0.130
Ovarian cancer	0.976	0.011	1.129	0.023	1.422	1.684
Parkinson's disease	1.127	0.066	1.865	0.142	1.410	2.245
Peptic ulcer disease	0.245	0.090	0.289	0.115	0.290	0.080
Pneumoconiosis	0.053	0.004	0.066	0.011	0.057	1.464
Prostate cancer	2.120	0.132	2.798	0.627	2.455	2.636
Schizophrenia	2.273	0.030	0.000	0.369	0.000	2.636
Sexually transmitted infections excluding HIV	0.109	9.575	0.010	14.980	0.014	3.549
Stroke	8.756	0.195	10.969	1.628	9.436	3.508
Tracheal, bronchus, and lung cancer	9.580	0.109	11.938	0.102	14.209	4.200
Tuberculosis	0.057	0.003	0.057	8.466	0.073	4.892
Uterine cancer	0.571	0.034	0.594	0.145	0.719	0.361

Percentage data values for 2019 (HIV/AIDS removed).

CAUSES-2019	DALYs	Incidence	Deaths	Prevalence	YLL	Funding
Alcohol use disorders	3.001	5.870	0.813	2.732	1.690	5.938
Alzheimer's disease and other dementias	4.769	1.048	8.332	1.648	4.613	23.924
Asthma	3.328	6.523	0.236	11.411	0.406	3.343
Breast cancer	3.302	0.401	3.186	0.947	4.177	7.572
Cervical cancer	0.529	0.030	0.463	0.037	0.743	1.132
Chronic obstructive pulmonary disease	11.815	2.082	11.338	6.771	10.681	1.196
Cirrhosis and other chronic liver diseases	4.296	0.143	3.896	15.824	6.242	3.749
Colon and rectum cancer	4.143	0.358	4.865	0.463	5.652	3.140
Diabetes mellitus	10.497	2.554	4.500	13.059	5.234	11.738
Idiopathic epilepsy	0.777	0.193	0.133	0.366	0.287	2.008
Ischemic heart disease	21.054	1.511	32.286	2.980	29.804	4.496
Major depressive disorder	5.276	26.031	0.000	3.799	0.000	1.399
Multiple sclerosis	0.497	0.016	0.239	0.138	0.383	1.186
Otitis media	0.118	15.444	0.002	0.865	0.003	0.139
Ovarian cancer	1.004	0.042	1.129	0.034	1.422	1.794
Parkinson's disease	1.158	0.242	1.865	0.208	1.410	2.392
Peptic ulcer disease	0.252	0.332	0.289	0.168	0.290	0.085
Pneumoconiosis	0.055	0.016	0.066	0.016	0.057	1.559
Prostate cancer	2.180	0.486	2.798	0.918	2.455	2.809
Schizophrenia	2.337	0.112	0.000	0.540	0.000	2.809
Sexually transmitted infections excluding HIV	0.112	35.308	0.010	21.935	0.014	3.781
Stroke	9.003	0.717	10.969	2.383	9.436	3.738
Tracheal, bronchus, and lung cancer	9.850	0.401	11.938	0.149	14.209	4.475
Tuberculosis	0.059	0.013	0.057	12.397	0.073	5.212
Uterine cancer	0.587	0.126	0.594	0.212	0.719	0.384

Percentage data values for 2019 (HIV/AIDS and Oral disorders removed).

Diseases-GROSS-1999 (1994 Burden & 1996 Funding)	DALYs	Incidence	Mortality	Prevalence	YLL	NIH FUNDING (%)
ALCOHOL ABUSE	7.492	11.457	0.470	12.275	0.773	5.211
Alzheimer's disease and other dementias (DEMENTIA)	4.578	0.991	2.285	4.805	0.781	6.182
ASTHMA	1.974	3.715	0.336	10.800	0.513	1.657
BREAST CANCER	2.270	0.541	2.957	1.535	3.361	7.755
CERVICAL CANCER	0.307	0.004	0.336	0.111	0.497	1.222
CHRONIC OBSTRUCTIVE PULMONARY DISEASE	3.648	0.692	6.452	2.898	4.087	1.267
CIRRHOSIS	2.530	0.174	2.084	0.840	3.077	3.448
COLORECTAL CANCER	2.597	0.516	3.898	1.307	3.085	2.143
DIABETES MELLITUS	3.765	2.385	3.831	25.680	3.211	6.070
HIV/AIDS	2.024	0.143	2.823	0.884	7.724	28.651
Idiopathic epilepsy (EPILEPSY)	0.599	0.521	0.067	3.061	0.197	1.119
ISCHEMIC HEART DISEASE	14.178	2.290	32.329	6.090	23.455	5.465
Major depressive disorder (DEPRESSION)	13.406	21.314	0.538	8.674	0.181	2.920
MULTIPLE SCLEROSIS	0.377	0.017	0.134	0.313	0.221	1.681
Oral disorders (DENTAL AND ORAL DISORDERS)	1.390	0.000	0.007	0.000	0.007	3.799
OTITIS MEDIA	0.013	41.780	0.003	2.110	0.006	0.185
OVARIAN CANCER	0.807	0.076	0.941	0.168	0.379	0.856
PARKINSON'S DISEASE	0.714	0.141	0.672	1.254	0.284	1.567
PEPTIC ULCER	0.382	0.976	0.403	3.396	0.276	0.122
Pneumoconiosis (PNEUMONIA)	2.017	9.567	5.444	0.121	2.864	1.257
PROSTATE CANCER	0.917	0.467	2.352	1.370	1.207	1.882
SCHIZOPHRENIA	3.592	0.167	0.027	4.861	0.016	2.264
SEXUALLY TRANSMITTED DISEASES	0.645	0.000	0.020	0.000	0.221	2.083
STROKE	7.950	1.325	10.283	6.423	5.885	2.443
Tracheal, bronchus, and lung cancer (LUNG CANCER)	4.771	0.444	10.015	0.593	9.136	2.595
TUBERCULOSIS	0.188	0.160	0.134	0.026	0.118	1.302
UTERINE CANCER	0.296	0.137	0.202	0.406	0.181	0.283

Raw subset of percentage data values from Gross et al, 1999 (all disorders).

Diseases-GROSS-1999 (1994 Burden & 1996 Funding)	DALYs	Incidence	Mortality	Prevalence	YLL	NIH FUNDING (%)
ALCOHOL ABUSE	8.980	11.457	0.528	12.275	1.078	5.460
Alzheimer's disease and other dementias (DEMENTIA)	5.487	0.991	2.566	4.805	1.089	6.478
ASTHMA	2.367	3.715	0.377	10.800	0.715	1.736
BREAST CANCER	2.721	0.541	3.321	1.535	4.685	8.126
CERVICAL CANCER	0.368	0.004	0.377	0.111	0.693	1.281
CHRONIC OBSTRUCTIVE PULMONARY DISEASE	4.373	0.692	7.246	2.898	5.696	1.328
CIRRHOSIS	3.033	0.174	2.340	0.840	4.289	3.613
COLORECTAL CANCER	3.113	0.516	4.378	1.307	4.300	2.246
DIABETES MELLITUS	4.513	2.385	4.302	25.680	4.476	6.361
HIV/AIDS	2.426	0.143	3.170	0.884	10.766	30.024
Idiopathic epilepsy (EPILEPSY)	0.718	0.521	0.075	3.061	0.275	1.173
ISCHEMIC HEART DISEASE	16.994	2.290	36.306	6.090	32.693	5.726
Major depressive disorder (DEPRESSION)	16.070	21.314	0.604	8.674	0.253	3.060
MULTIPLE SCLEROSIS	0.452	0.017	0.151	0.313	0.308	1.762
Oral disorders (DENTAL AND ORAL DISORDERS)	1.666	0.000	0.008	0.000	0.010	3.981
OTITIS MEDIA	0.015	41.780	0.003	2.110	0.008	0.194
OVARIAN CANCER	0.967	0.076	1.057	0.168	0.528	0.897
PARKINSON'S DISEASE	0.856	0.141	0.755	1.254	0.396	1.642
PEPTIC ULCER	0.458	0.976	0.453	3.396	0.385	0.128
Pneumoconiosis (PNEUMONIA)	2.418	9.567	6.114	0.121	3.992	1.317
PROSTATE CANCER	1.099	0.467	2.642	1.370	1.683	1.972
SCHIZOPHRENIA	4.306	0.167	0.030	4.861	0.022	2.372
SEXUALLY TRANSMITTED DISEASES	0.774	0.000	0.023	0.000	0.308	2.183
STROKE	9.529	1.325	11.549	6.423	8.204	2.560
Tracheal, bronchus, and lung cancer (LUNG CANCER)	5.719	0.444	11.247	0.593	12.734	2.719
TUBERCULOSIS	0.226	0.160	0.151	0.026	0.165	1.365
UTERINE CANCER	0.354	0.137	0.226	0.406	0.253	0.297

Comparable percentage data values from Gross et al, 1999 (all disorders).

Diseases-GROSS-1999 (1994 Burden & 1996 Funding)	DALYs	Incidence	Mortality	Prevalence	YLL	NIH FUNDING (%)
ALCOHOL ABUSE	9.203	11.473	0.546	12.384	1.208	7.803
Alzheimer's disease and other dementias (DEMENTIA)	5.624	0.993	2.650	4.848	1.220	9.257
ASTHMA	2.425	3.720	0.390	10.897	0.801	2.481
BREAST CANCER	2.788	0.541	3.430	1.548	5.250	11.613
CERVICAL CANCER	0.377	0.004	0.390	0.112	0.776	1.830
CHRONIC OBSTRUCTIVE PULMONARY DISEASE	4.482	0.693	7.483	2.924	6.384	1.898
CIRRHOSIS	3.108	0.174	2.417	0.847	4.806	5.164
COLORECTAL CANCER	3.191	0.516	4.521	1.318	4.818	3.209
DIABETES MELLITUS	4.625	2.389	4.443	25.909	5.016	9.090
Idiopathic epilepsy (EPILEPSY)	0.736	0.522	0.078	3.088	0.308	1.676
ISCHEMIC HEART DISEASE	17.417	2.294	37.495	6.144	36.638	8.183
Major depressive disorder (DEPRESSION)	16.469	21.344	0.624	8.752	0.283	4.373
MULTIPLE SCLEROSIS	0.463	0.017	0.156	0.316	0.345	2.518
Oral disorders (DENTAL AND ORAL DISORDERS)	1.707	0.000	0.008	0.000	0.011	5.690
OTITIS MEDIA	0.016	41.839	0.003	2.129	0.009	0.277
OVARIAN CANCER	0.991	0.077	1.091	0.169	0.592	1.282
PARKINSON'S DISEASE	0.877	0.141	0.780	1.266	0.444	2.346
PEPTIC ULCER	0.469	0.977	0.468	3.426	0.431	0.182
Pneumoconiosis (PNEUMONIA)	2.478	9.581	6.314	0.122	4.473	1.882
PROSTATE CANCER	1.126	0.468	2.728	1.383	1.885	2.818
SCHIZOPHRENIA	4.413	0.168	0.031	4.904	0.025	3.390
SEXUALLY TRANSMITTED DISEASES	0.793	0.000	0.023	0.000	0.345	3.120
STROKE	9.766	1.327	11.927	6.480	9.193	3.658
Tracheal, bronchus, and lung cancer (LUNG CANCER)	5.861	0.445	11.615	0.598	14.271	3.886
TUBERCULOSIS	0.232	0.160	0.156	0.027	0.185	1.950
UTERINE CANCER	0.363	0.138	0.234	0.410	0.283	0.424

Comparable percentage data values from Gross et al, 1999 (HIV/AIDS removed).

Diseases-GROSS-1999 (1994 Burden & 1996 Funding)	DALYs	Incidence	Mortality	Prevalence	YLL	NIH FUNDING (%)
ALCOHOL ABUSE	9.363	11.473	0.546	12.384	1.208	8.274
Alzheimer's disease and other dementias (DEMENTIA)	5.721	0.993	2.651	4.848	1.220	9.816
ASTHMA	2.467	3.720	0.390	10.897	0.801	2.631
BREAST CANCER	2.837	0.541	3.430	1.548	5.250	12.313
CERVICAL CANCER	0.383	0.004	0.390	0.112	0.776	1.940
CHRONIC OBSTRUCTIVE PULMONARY DISEASE	4.560	0.693	7.484	2.924	6.384	2.012
CIRRHOSIS	3.162	0.174	2.417	0.847	4.807	5.475
COLORECTAL CANCER	3.246	0.516	4.522	1.318	4.819	3.403
DIABETES MELLITUS	4.705	2.389	4.444	25.909	5.016	9.638
Idiopathic epilepsy (EPILEPSY)	0.749	0.522	0.078	3.088	0.308	1.777
ISCHEMIC HEART DISEASE	17.719	2.294	37.498	6.144	36.642	8.677
Major depressive disorder (DEPRESSION)	16.755	21.344	0.624	8.752	0.283	4.637
MULTIPLE SCLEROSIS	0.471	0.017	0.156	0.316	0.345	2.670
OTITIS MEDIA	0.016	41.839	0.003	2.129	0.009	0.293
OVARIAN CANCER	1.008	0.077	1.091	0.169	0.592	1.360
PARKINSON'S DISEASE	0.892	0.141	0.780	1.266	0.444	2.488
PEPTIC ULCER	0.477	0.977	0.468	3.426	0.431	0.193
Pneumoconiosis (PNEUMONIA)	2.521	9.581	6.315	0.122	4.474	1.996
PROSTATE CANCER	1.146	0.468	2.729	1.383	1.886	2.988
SCHIZOPHRENIA	4.490	0.168	0.031	4.904	0.025	3.595
SEXUALLY TRANSMITTED DISEASES	0.807	0.000	0.023	0.000	0.345	3.308
STROKE	9.936	1.327	11.928	6.480	9.194	3.878
Tracheal, bronchus, and lung cancer (LUNG CANCER)	5.963	0.445	11.616	0.598	14.272	4.121
TUBERCULOSIS	0.236	0.160	0.156	0.027	0.185	2.068
UTERINE CANCER	0.369	0.138	0.234	0.410	0.283	0.450

Comparable percentage data values from Gross et al, 1999 (HIV/AIDS and Oral disorders removed).

Diseases-Gillum 2011 (Burden 2004, Funding 2006)	DALYs	Incidence	Mortality	Prevalence	YLL	NIH Funding in Millions
Alcohol abuse	6.697	1.637	0.448	7.253	0.929	4.296
Alzheimer's disease and other dementias (dementia)	4.955	0.443	7.395	2.360	2.349	5.405
Asthma	2.753	0.792	0.224	14.501	0.407	2.379
Breast cancer	2.494	0.138	2.969	1.424	3.746	6.036
Cervical cancer	0.456	0.018	0.392	0.171	0.660	0.815
Chronic obstructive pulmonary disorder	6.005	0.266	7.395	5.256	4.944	0.563
Cirrhosis	1.659	0.027	1.681	0.230	2.764	3.430
Colorectal cancer	2.220	0.093	3.922	0.541	3.577	2.261
Diabetes mellitus	5.370	0.744	4.706	16.447	4.322	8.726
HIV/AIDS	2.125	0.088	0.784	0.968	2.142	24.395
Idiopathic epilepsy (epilepsy)	0.583	0.128	0.112	1.273	0.238	0.866
Ischemic heart disease	11.112	0.828	29.748	1.782	20.689	3.346
Major depressive disorder (depression)	16.639	10.175	0.056	6.231	0.023	2.816
Multiple sclerosis	0.430	0.006	0.224	0.134	0.345	0.925
Oral disorders (Dental and oral disorders)	0.973	68.038	0.000	31.244	0.015	3.472
Otitis media	0.128	10.957	0.000	1.033	0.008	0.143
Ovarian cancer	0.587	0.015	0.952	0.109	1.075	0.857
Parkinson's disease	0.959	0.056	1.176	0.778	0.507	1.748
Peptic ulcer disease	0.146	0.119	0.224	0.541	0.184	0.143
Pneumonia	1.148	2.590	3.810	0.057	2.257	2.951
Prostate cancer	0.922	0.092	2.129	0.784	1.167	2.925
Schizophrenia	1.903	0.026	0.056	1.185	0.038	3.060
Sexually transmitted diseases	0.237	0.000	0.000	0.000	0.015	2.219
Stroke	4.871	0.231	9.860	2.075	6.072	2.875
Tracheal, bronchus, and lung cancer (LUNG CANCER)	5.046	0.121	10.140	0.536	10.218	2.236
Tuberculosis	0.036	0.007	0.056	0.008	0.054	1.261
Uterine cancer	0.306	0.015	0.392	0.229	0.345	0.235

Raw subset of raw data values from Gillum et al, 2011 (all disorders).

Diseases-Gillum 2011 (Burden 2004, Funding 2006)	DALYs	Incidence	Mortality	Prevalence	YLL	NIH Funding in Millions
Alcohol abuse	8.293	1.676	0.504	7.466	1.344	4.753
Alzheimer's disease and other dementias (dementia)	6.135	0.453	8.323	2.429	3.400	5.980
Asthma	3.408	0.811	0.252	14.927	0.589	2.632
Breast cancer	3.088	0.141	3.342	1.465	5.422	6.678
Cervical cancer	0.564	0.018	0.441	0.176	0.956	0.902
Chronic obstructive pulmonary disorder	7.435	0.272	8.323	5.410	7.156	0.623
Cirrhosis	2.054	0.027	1.892	0.237	4.000	3.795
Colorectal cancer	2.749	0.095	4.414	0.557	5.178	2.502
Diabetes mellitus	6.650	0.762	5.296	16.930	6.256	9.654
HIV/AIDS	2.632	0.090	0.883	0.996	3.100	26.990
Idiopathic epilepsy (epilepsy)	0.722	0.131	0.126	1.311	0.344	0.958
Ischemic heart disease	13.759	0.848	33.480	1.834	29.944	3.702
Major depressive disorder (depression)	20.603	10.420	0.063	6.414	0.033	3.116
Multiple sclerosis	0.533	0.006	0.252	0.138	0.500	1.023
Oral disorders (Dental and oral disorders)	1.205	69.676	0.000	32.161	0.022	3.841
Otitis media	0.158	11.221	0.000	1.063	0.011	0.158
Ovarian cancer	0.727	0.015	1.072	0.112	1.556	0.949
Parkinson's disease	1.187	0.057	1.324	0.801	0.733	1.935
Peptic ulcer disease	0.181	0.122	0.252	0.556	0.267	0.158
Pneumonia	1.422	2.652	4.288	0.059	3.267	3.265
Prostate cancer	1.142	0.095	2.396	0.807	1.689	3.237
Schizophrenia	2.356	0.027	0.063	1.220	0.056	3.385
Sexually transmitted diseases	0.293	0.000	0.000	0.000	0.022	2.455
Stroke	6.031	0.237	11.097	2.136	8.789	3.181
Tracheal, bronchus, and lung cancer (LUNG CANCER)	6.248	0.124	11.412	0.552	14.789	2.474
Tuberculosis	0.045	0.007	0.063	0.009	0.078	1.395
Uterine cancer	0.379	0.016	0.441	0.235	0.500	0.260

Comparable percentage data values from Gillum et al, 2011 (all disorders).

Diseases-Gillum 2011 (Burden 2004, Funding 2006)	DALYs	Incidence	Mortality	Prevalence	YLL	NIH Funding in Millions
Alcohol abuse	8.517	1.678	0.509	7.541	1.387	6.510
Alzheimer's disease and other dementias (dementia)	6.301	0.454	8.397	2.453	3.509	8.191
Asthma	3.500	0.812	0.254	15.077	0.608	3.605
Breast cancer	3.171	0.141	3.372	1.480	5.596	9.146
Cervical cancer	0.580	0.018	0.445	0.178	0.986	1.236
Chronic obstructive pulmonary disorder	7.636	0.273	8.397	5.465	7.384	0.854
Cirrhosis	2.110	0.027	1.908	0.239	4.128	5.197
Colorectal cancer	2.823	0.095	4.453	0.563	5.343	3.427
Diabetes mellitus	6.829	0.762	5.344	17.100	6.456	13.223
Idiopathic epilepsy (epilepsy)	0.742	0.132	0.127	1.324	0.355	1.312
Ischemic heart disease	14.131	0.849	33.779	1.853	30.902	5.070
Major depressive disorder (depression)	21.160	10.430	0.064	6.478	0.034	4.268
Multiple sclerosis	0.547	0.006	0.254	0.139	0.516	1.401
Oral disorders (Dental and oral disorders)	1.238	69.739	0.000	32.485	0.023	5.261
Otitis media	0.162	11.231	0.000	1.074	0.011	0.217
Ovarian cancer	0.746	0.015	1.081	0.113	1.605	1.299
Parkinson's disease	1.219	0.057	1.336	0.809	0.757	2.650
Peptic ulcer disease	0.185	0.122	0.254	0.562	0.275	0.217
Pneumonia	1.460	2.654	4.326	0.059	3.371	4.471
Prostate cancer	1.173	0.095	2.417	0.815	1.743	4.433
Schizophrenia	2.420	0.027	0.064	1.232	0.057	4.637
Sexually transmitted diseases	0.301	0.000	0.000	0.000	0.023	3.363
Stroke	6.194	0.237	11.196	2.157	9.070	4.357
Tracheal, bronchus, and lung cancer (LUNG CANCER)	6.417	0.125	11.514	0.557	15.262	3.389
Tuberculosis	0.046	0.007	0.064	0.009	0.080	1.911
Uterine cancer	0.389	0.016	0.445	0.238	0.516	0.357

Comparable percentage data values from Gillum et al, 2011 (HIV/AIDS removed).

Diseases-Gillum 2011 (Burden 2004, Funding 2006)	DALYs	Incidence	Mortality	Prevalence	YLL	NIH Funding in Millions
Alcohol abuse	8.624	5.544	0.509	11.169	1.388	6.871
Alzheimer's disease and other dementias (dementia)	6.380	1.499	8.397	3.634	3.510	8.646
Asthma	3.544	2.683	0.254	22.332	0.608	3.805
Breast cancer	3.211	0.466	3.372	2.192	5.597	9.654
Cervical cancer	0.587	0.061	0.445	0.263	0.986	1.304
Chronic obstructive pulmonary disorder	7.732	0.901	8.397	8.094	7.386	0.901
Cirrhosis	2.136	0.090	1.908	0.354	4.129	5.486
Colorectal cancer	2.859	0.315	4.453	0.834	5.345	3.617
Diabetes mellitus	6.915	2.519	5.344	25.328	6.457	13.957
Idiopathic epilepsy (epilepsy)	0.751	0.435	0.127	1.961	0.356	1.385
Ischemic heart disease	14.309	2.805	33.779	2.744	30.910	5.352
Major depressive disorder (depression)	21.425	34.465	0.064	9.596	0.034	4.505
Multiple sclerosis	0.554	0.019	0.254	0.206	0.516	1.479
Otitis media	0.164	37.114	0.000	1.590	0.011	0.229
Ovarian cancer	0.756	0.050	1.081	0.167	1.606	1.372
Parkinson's disease	1.235	0.189	1.336	1.198	0.757	2.797
Peptic ulcer disease	0.188	0.403	0.254	0.832	0.275	0.229
Pneumonia	1.479	8.771	4.326	0.088	3.372	4.720
Prostate cancer	1.188	0.313	2.417	1.207	1.743	4.679
Schizophrenia	2.450	0.088	0.064	1.825	0.057	4.894
Sexually transmitted diseases	0.305	0.000	0.000	0.000	0.023	3.550
Stroke	6.272	0.783	11.196	3.195	9.072	4.599
Tracheal, bronchus, and lung cancer (LUNG CANCER)	6.497	0.411	11.514	0.825	15.266	3.577
Tuberculosis	0.047	0.023	0.064	0.013	0.080	2.017
Uterine cancer	0.394	0.052	0.445	0.352	0.516	0.376

Comparable percentage data values from Gillum et al, 2011 (HIV/AIDS and Oral disorders removed).

References

Gillum LA, Gouveia C, Dorsey ER, et al. NIH disease funding levels and burden of disease. *PLoS One*. 2011;6(2): e16837. doi:10.1371/journal.pone.0016837

Gross CP, Anderson GF, Powe NR. The relation between funding by the National Institutes of Health and the burden of disease. *N Engl J Med*. 1999;340(24):1881-1887. doi:10.1056/NEJM199906173402406
